# Extracellular matrix and Hippo signaling as therapeutic targets of antifibrotic compounds for uterine fibroids

**DOI:** 10.1002/ctm2.475

**Published:** 2021-07-04

**Authors:** Md Soriful Islam, Sadia Afrin, Bhuchitra Singh, Friederike L. Jayes, Joshua T. Brennan, Mostafa A. Borahay, Phyllis C. Leppert, James H. Segars

**Affiliations:** ^1^ Department of Gynecology and Obstetrics, Division of Reproductive Sciences & Women's Health Research Johns Hopkins Medicine Baltimore Maryland USA; ^2^ Department of Obstetrics and Gynecology Duke University Durham North Carolina USA

**Keywords:** collagenase, extracellular matrix, Hippo signaling, nintedanib, uterine fibroids, verteporfin

## Abstract

**Background:**

Uterine fibroids are highly prevalent, collagen‐rich, mechanically stiff, fibrotic tumors for which new therapeutic options are needed. Increased extracellular matrix (ECM) stiffness activates mechanical signaling and Hippo/YAP promoting fibroid growth, but no prior studies have tested either as a therapeutic target. We tested the hypothesis that injection of a purified form of collagenase *Clostridium histolyticum* (CCH) that selectively digests type I and type III collagens would alter ECM stiffness, Hippo signaling, and selectively reduce fibroid cell growth. We also used two FDA‐approved drugs, verteporfin and nintedanib, to elucidate the role of Hippo/YAP signaling in uterine fibroid and myometrial cells.

**Methods:**

The clinical trial was registered (NCT02889848). Stiffness of samples was measured by rheometry. Protein expression in surgical samples was analyzed via immunofluorescence. Protein and gene expression in uterine fibroid or myometrial cell lines were measured by real time PCR and western blot, and immunofluorescence.

**Results:**

Injection of CCH at high doses (0.1–0.2 mg/cm^3^) into fibroids resulted in a 46% reduction in stiffness in injected fibroids compared to controls after 60 days. Levels of the cell proliferation marker proliferative cell nuclear antigen (PCNA) were decreased in fibroids 60 days after injection at high doses of CCH. Key Hippo signaling factors, specifically the transcriptionally inactive phosphorylated YAP (p‐YAP), was increased at high CCH doses, supporting the role of YAP in fibroid growth. Furthermore, inhibition of YAP via verteporfin (YAP inhibitor) decreased cell proliferation, gene and protein expression of key factors promoting fibrosis and mechanotransduction in fibroid cells. Additionally, the anti‐fibrotic drug, nintedanib, inhibited YAP and showed anti‐fibrotic effects.

**Conclusions:**

This is the first report that *in vivo* injection of collagenase into uterine fibroids led to a reduction in Hippo/YAP signaling and crucial genes and pathways involved in fibroid growth. These results indicate that targeting ECM stiffness and Hippo signaling might be an effective strategy for uterine fibroids.

## Graphical abstract

Inactivation of YAP signaling by anti‐fibrotic compounds in uterine fibroid cells. Abbreviations: ECM, extracellular matrix; *CTGF*, connective tissue growth factor; *FN1*, fibronectin; *INHBA*, inhibin subunit beta A; P, phosphorylation; *PAI‐1*, plasminogen activator inhibitor 1; TEAD, TEA domain family member; *VCAN*, versican; YAP, yes‐associated protein.

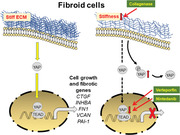

## 1 INTRODUCTION

Uterine fibroids are a common benign tumor of the uterus. The estimated prevalence of uterine fibroids exceeds 80% in black women and approaches 70% in white women by age 50.^1^ Clinical symptoms occur in 20%–50% of women with fibroids^1^, ^2^ making fibroid disease among the most frequently encountered health conditions of women. Women with fibroids consider the impact of the disease on their life similar to a diagnosis of diabetes, heart disease, or breast cancer.^3^ Many women with symptomatic uterine fibroids require medical or surgical interventions. Medical treatments for fibroids remain limited.^4^, ^5^ Gonadotrophin‐releasing hormone agonist (GnRHa)^6^ and antagonists (GnRHant),^7^ and selective progesterone receptor modulators (such as ulipristal acetate)^8^ reduce abnormal uterine bleeding, but only temporarily reduce fibroid size. Moreover, the association of GnRHa and GnRHant with menopausal symptoms and bone loss^6^, ^9^ and rare but serious liver injury with ulipristal acetate^10^ limits their use. The use of ulipristal acetate for uterine fibroid treatment in the United States remains uncertain. Surgical treatments are associated with risks and negatively impact on women's quality of life.^3^ Furthermore, the annual cost associated with fibroid management is estimated to be between $5.9 and $34.4 billion (2010 USD) in the United States.^11^ Therefore, there is a pressing need for non‐surgical treatment alternatives.

While the molecular mechanisms of fibroid development and growth remain poorly understood, these tumors represent a fascinating model to explore tumor behavior because the tumors grow to several centimeters (7–10 centimeter tumors are common) and affect surrounding tissues, but only very rarely do they metastasize. Genetic analysis shows fibroid‐tumors originate from a clonal expansion of single smooth muscle progenitor/stem cells, each having a single distinct mutation, such as *MED12*, suggesting a role of genetics in fibroid development and growth.^12^ Furthermore, epigenetic changes can also occur.^13^ While the cause of uterine fibroids remains unclear, recent research has implicated a role for the Hippo signaling pathway, a signaling pathway that integrates mechanical signaling with cell growth.

A cardinal feature of uterine fibroids is the presence of an excessive amount of extracellular matrix (ECM), including collagens.^14^, ^15^ Collagens in fibroids are primarily types I and III, with widely dispersed non‐parallel short collagen fibrils, which contributes to fibroid stiffness and their massive size.^15^, ^16^, ^17^, ^18^ Increased ECM stiffness is known to mechanoactivate YAP/TAZ (TAZ is a paralogue of YAP), which promotes the transcription of cell growth and fibrotic genes.^19^, ^20^, ^21^, ^22^ In a soft matrix, the Hippo pathway is turned "ON," YAP/TAZ then becomes phosphorylated or degraded in the cytoplasm, thus preventing the translocation of YAP/TAZ into the nucleus.^19^, ^23^ YAP/TAZ are overexpressed in cancers and fibrosis of many organs, including liver, lung, and kidney.^24^ Recently, we and others demonstrated that YAP/TAZ were overexpressed in uterine fibroids.^25^, ^26^ Collectively, these observations led us to test the hypothesis that manipulation of the Hippo pathway (phosphorylated YAP) might be an effective strategy to control or reduce fibroid growth.

To test this hypothesis, we studied three US FDA‐approved drugs that are used for treatment of pathologic fibrotic conditions but that have not been tested for effects on uterine fibroids. The drugs include collagenase *Clostridium histolyticum* (CCH), verteporfin, and nintedanib. CCH is an enzyme isolated from bacterium CCH that is used for treatment of Dupuytren's contracture^27^ and for Peyronie's disease,^28^ and is being studied for other conditions, such as adhesive capsulitis, lipoma, and cellulite. CCH is known to degrade type I and type III collagens,^29^ which are overexpressed in uterine fibroids.^30^ Of note, leakage into surrounding tissues has not been a problem, likely because this form of collagenase does not digest collagens of vessels and nerves and is rapidly inactivated by serum proteins. Ex vivo treatment of collagen‐rich uterine fibroid tissues with CCH for 96 h resulted in substantially reduced fibrosis.^31^ The reduction in fibrosis by CCH treatment was associated with digestion of collagen fibrils and reduction of tissue stiffness.^31^, ^32^ Verteporfin was selected as a known inhibitor of YAP^33^, ^34^ to explore the role of Hippo signaling in fibroid cells. Verteporfin was identified by screening 3300 drugs from Johns Hopkins Drug Library and is a specific inhibitor of YAP‐TEAD (DNA binding partner of YAP/TAZ) interactions.^34^ This drug is used in photodynamic therapy to eliminate abnormal blood vessels in the eye associated with conditions such as macular degeneration. Verteporfin also exerts antifibrotic effects in Dupuytren's disease^35^ and oral submucous fibrosis in mice.^36^ In addition, we hypothesized that antifibrotic drug, nintedanib,^37^ might be effective in regulating expression of genes involved in fibrosis in fibroids involving Hippo/YAP signaling. Nintedanib is an inhibitor of three tyrosine kinases, PDGFR, FGFR, and VEGFR. This drug is used for the treatment of idiopathic pulmonary fibrosis^37^ and systemic sclerosis‐associated interstitial lung disease (SSc‐ILD).^38^ Nintedanib was also effective in attenuating CCL4 (carbon tetrachloride)‐induced pathology and reducing hepatic injury, inflammation, and fibrosis in a mouse model.^39^ In addition, in the EU countries, nintedanib plus docetaxel is used for patients with advanced non‐small cell lung cancer after first‐line chemotherapy. Our first aim was to test the hypothesis that collagenase injection into uterine fibroids would alter tissue stiffness and markers of cell growth and Hippo signaling. Our second aim was to explore whether Hippo signaling proteins, such as YAP, a transcriptional effector of Hippo signaling, could be used to target fibroid growth by the utilization of the YAP inhibitor, verteporfin, and the antifibrotic drug, nintedanib. Understanding the mechanisms of actions of these three drugs against active pathophysiological processes involved in fibroid growth, fibrosis, and mechanotransduction may support their further exploration as ‐ re‐purposed novel medical therapies for fibroids.

## 2 MATERIALS AND METHODS

### 2.1 Study design

The tissues were obtained in an open‐label, Phase 1, dose‐escalation pilot study (ClinicalTrials.gov number NCT02889848) of CCH included premenopausal women with symptomatic uterine fibroids (*n* = 12) undergoing hysterectomy at Johns Hopkins Hospital, Baltimore, MD, USA. All patients provided signed, informed consent. The study protocol and all procedures were approved by Institutional Review Board at Johns Hopkins University School of Medicine. The ratio of black to white study subjects was 3:2 with an age range of 35‐ to 50‐year‐old. Injected and control fibroids were from the same patient and were thus patient‐matched. The location of the fibroids was intramural with diameters of 3–10 cm. Interim hormonal treatment until hysterectomy was allowed (only one subject received hormonal treatment while being enrolled in the study). Women with BMI > 40 kg/m,^2^ history of allergic reaction to collagenase (specifically EN3835, a highly purified preparation of collagenase), cancer within the past 5 years, abnormal liver function test (>20% elevation), severe anemia (HCT < 30), recent rapid growth of fibroids, and submucosal (FIGO type 0) and subserosal pedunculated (FIGO type 7) fibroids were excluded from this study.

**HIGHLIGHTS**

- Collagenase injection softened extracellular matrix stiffness and altered Hippo signaling by increasing the transcriptionally inactive form of YAP (phospho‐YAP).
- Verteporfin and nintedanib inactivated YAP and reduced transcription of genes involved in fibroid cell growth and fibrosis.
- The results suggest a possible role of Hippo/YAP signaling as a therapeutic target for fibroid treatment.

### 2.2 CCH injection

Fibroids exhibit heterogeneity in size and cellularity. To avoid injection into cancerous fibroids, or syndromic fibroids at risk for cancer (for instance hereditary diseases such as hereditary leiomyomatosis and renal cell cancer) we only injected fibroids with an MRI signature suggesting common fibroids (T2‐weighted hypointense signal). This was a dose‐finding study, and the dose of collagenase was determined by the size of the fibroid as shown (Table S1). Fibroids were injected under ultrasound guidance via a transvaginal route under sedation. CCH was slowly injected into the central region of the fibroid. The injected CCH was visualized under ultrasound. For large fibroids, the needle was repositioned (not withdrawn entirely), and several injections in the center were achieved (Figures S1, S2, and S3). A fixed dose (1.16 mg) of CCH was injected into fibroids from three patients (*n* = 3) or left untreated (control; *n* = 3), and hysterectomy was performed after 24–48 h to test for possible complications as this was the first time collagenase had been injected into fibroids. In each patient, one fibroid was injected with CCH, and one fibroid was untreated (control). The remaining nine patients were divided into three groups of increasing dosage. Fibroids were injected with CCH at 0.05 mg/cm^3^ (dose 1; *n* = 3) or left untreated (control; *n* = 3), 0.1 mg/cm^3^ (dose 2; *n* = 3) or let untreated (control; *n* = 3), and 0.2 mg/cm^3^ (dose 3; *n* = 3), or untreated (control; *n* = 3). Hysterectomies were performed after 60–90 days for these nine patients. CCH dose was selected based on ex‐vivo studies^31^ and previously approved dosing in Dupuytren's disease. Therefore, the maximum amount of CCH for a single fibroid was 1.68, 3.35, and 5.028 mg for doses 1, 2, and 3, respectively. Each 1 cm^3^ of fibroid volume received approximately 50–70 μl of CCH. Effects of CCH were localized to the central region of the fibroids injected and were apparent by gross examination at the time of removal by palpation and appearance. Areas analyzed by rheometry focused on these areas. Gross changes could be observed in the injected fibroids (Figure S3). The injected fibroid was noticeably less rigid on palpation as compared to the control fibroid. There was a visible digestion of tissue observed on sectioning the fibroid (FIB014). Details of the study and technique and side effects are reported elsewhere.^40^

### 2.3 Tissue preparation

Fibroids treated with CCH or untreated (control) were excised from women undergoing hysterectomy for fibroids. The samples were hemisected to expose the center of the fibroid. A part of each fibroid was immediately frozen and stored at ‐80°C for stiffness analysis by rheometry, and another part was formalin fixed and paraffin embedded. Paraffin blocks were sectioned in 5 μm slices for immunofluorescence analysis.

### 2.4 Rheometry analysis

To quantify the stiffness of CCH‐treated and control fibroid tissues, we conducted rheometry analysis adapted from the previously described procedure.^31^, ^32^, ^41^ The tissues were frozen to facilitate cutting. Using an 8 mm diameter punch, 1 or 2 tissue cores were obtained from each fibroid, trimmed to 2 mm height, and a strain sweep was performed from 0.1%–1.0 % horizontal strain on each core to ensure linearity at the selected strain. Frequency sweeps (0.5–50 rad/s) were performed with 20% vertical strain and 0.2% horizontal strain, and the complex shear modulus (G*) in [Pa] at 7 rad/s was reported.

### 2.5 Cell culture

Human myometrial (P51M) and fibroid (P51F) cells (courtesy of Minnie Malik, PhD; Uniformed Services University of the Health Sciences, Bethesda, MD) were cultured in fresh DMEM‐F12 supplemented with 10% FBS (GIBCO by Life Technologies) and 100 μg/L of normocin (InvivoGen, CA, USA) at 37°C in 95% air‐5% CO_2_. P51M and P51F cells were immortalized by HPV‐16 (Human papillomavirus 16).^42^ Cells were isolated from patient matched fibroid and myometrial tissues. As a representative model of fibroid and myometrial cells, a number of reports have been published using this cell lines.^43^, ^44^, ^45^ For immunofluorescence studies, myometrial and fibroid cells were cultured in 4 or 8 well chamber slides to reach 70% confluency and then serum starved (no serum) for 24 h. Starvation media was replaced with DMEM‐F12 supplemented with FBS and normocin and incubated at 37°C in 95% air‐5% CO_2_ for 24 h. Next, cells were fixed with 4% formaldehyde diluted in 1X PBS for 15 min at room temperature. For real time qPCR and western blot analysis, myometrial and fibroid cells were cultured in 60 mm or 100 mm dishes to reach 70% confluency and then serum starved (no serum) for 24 h. Next day, cells were treated (complete media) with verteporfin at 1 μM or nintedanib at 5 μM, or with vehicle control (DMSO) for 24 h.

### 2.6 Immunofluorescence

Paraffin sections of fibroid tissues were deparaffinized via xylene and rehydrated with a graded series of ethanol. Antigen retrieval was done using antigen unmasking solution (Vector laboratories #H3300) with a vegetable steamer at 95°C for 10 min following cooldown at room temperature for 30 min. Fixed tissue and cultured cells were then washed three times (5 min for each) with 1X PBS. To block nonspecific background, cells were incubated with blocking buffer (1X PBS/5% Normal Goat Serum/0.3% Triton X‐100) for 60 min. Fixed tissue or cells were then incubated overnight at 4°C with primary antibodies/antisera (Table S2) in antibody dilution buffer (1X PBS/1% BSA/0.3% Triton X‐100). Next day, cells were washed three times (5 min for each) in 1X PBS and incubated cells in fluorochrome‐conjugated secondary antibody Alexa Fluor 568 Goat anti‐rabbit IgG (H+L) (2 mg/ml) (A11036, Invitrogen) or Alexa Fluor 488 Goat anti‐mouse IgG (H+L) (2 mg/ml) (A‐11029, Invitrogen) diluted in antibody dilution buffer for 2 h at room temperature in the dark. Cells were washed three times (5 min for each) in 1X PBS and coverslipped with ProLong Gold Antifade Reagent with DAPI (8961, Cell Signaling Technology) and allowed mountant to dry overnight at room temperature under dark condition. Pictures were taken at 20X magnification with Zeiss AxioPlan 2 microscope system (Jena, Germany).

Negative controls were performed by omitting the first or the secondary antibody.

### 2.7 Cell proliferation assay

Myometrial and fibroid cells were seeded in 96‐well plates at initial densities of 2000 or 4000 cells/well and incubated at 37°C in 95% air‐5% CO_2_. Next day, cells were treated with verteporfin at different concentrations (0.1, 0.5, 1, 2, and 5 μM) for 24 h, or cells were treated with nintedanib at different concentrations (0.1, 0.5, 1, 5, and 10 μM) for 24 or 48 h. Cells treated with DMSO were used as a vehicle control. At the end of treatment, 20 μl of CellTiter 96 Aqueous One Solution Reagent (G3588, Promega) was directly added to each culture well and incubated for 2–3 h at 37°C with 5% CO_2,_ and then recorded the absorbance at 490 nm.

### 2.8 Real time qPCR

Cells were lysed with RLT buffer and purified with an RNeasy Mini Kit (Qiagen, Gaithersburg, MD). The concentration and purity of RNA were measured with the NanoVue Plus (Biochrom US, Holliston, MA, USA). The RNA was converted to cDNA (50‐100 ng/μl) using iScript cDNA Synthesis Kit (Bio‐Rad, Hercules, CA, USA) in a Bio‐Rad Thermocycler machine. The real time qPCR was performed on LightCycler 96 System (Roche Diagnostics, Mannheim, Germany) in 96‐well plate with 2–4 ng/μl of cDNA in a final volume of 10 μl containing 1X FastStart Essential DNA green Master (Roche Diagnostics), with appropriate primer sets of selected genes (Table S3). Primers were purchased from IDT (Coralville, IA, USA). The house keeping gene *RPLP0* was amplified under the same conditions for normalizing quantitative data. The relative mRNA expression was calculated using the ΔΔCT method and is presented as fold increase or decrease relative to control.

### 2.9 Western blot

Cells were washed with PBS and lysed with RIPA buffer (Sigma #R0278) containing protease and phosphatase inhibitor cocktail (Thermo Fisher Scientific). Protein concentrations were quantified using Pierce BCA Protein Assay Kit (Thermo Fisher Scientific). The equal volume (20–30 μg) of protein lysates was loaded onto 4%–12% NuPAGE gels (Thermo Fisher Scientific) and resolved by SDS‐PAGE under reducing conditions, and then transferred to 0.2‐μm nitrocellulose membranes in an X‐cell II apparatus (Thermo Fisher Scientific). Ponceau S solution (P7170, Sigma) was used for the detection of protein on nitrocellulose membranes. After blocking membranes with 5% non‐fat‐milk with TBST (1X TBS, 0.1% Tween 20) for 1 h, membranes were incubated overnight at 4°C with primary antibodies/antisera (Table S2). Then membranes were washed three times (5 min for each) with TBST and then incubated with appropriate horseradish peroxidase‐conjugated secondary antibodies as 1: 10,000 dilutions (NA934V or NA931V, GE Healthcare) with 5% non‐fat‐milk with TBST for 2 h at room temperature. Next, membranes were washed three times (5 min for each) with TBST, and immunoreactive proteins were visualized using a SuperSignal West Pico PLUS Chemiluminescent Substrate (Thermo Fisher Scientific) in an Azure Imager c300 system (Azure Biosystems, Dublin, CA, USA). The band intensity was quantified using Java‐based image processing program, ImageJ 1.52a and normalized against corresponding anti‐β‐actin.

### 2.10 Immunofluorescence image analysis

Random five images were captured at 20X (Tiff format) from CCH or untreated (control) slides using a Nikon Y‐THS microscope (Japan). Quantitative morphometric analysis was performed as previously reported.^46^ Protein expression was quantified by measuring the area and intensity of specific antibody staining using ImageJ software (version 1.52r; National Institutes of Health, USA). The Tiff images were opened in Image J and the scale set as distance in pixels: 97, known distance: 50, pixel aspect ratio: 1.0 and unit of length: μm. Tiff images were converted into an RGB stack, and then the threshold levels were adjusted. The integrated density (IntDen), area, area fraction, and limit to threshold were selected as parameters. Then, clicked on “measure” from “analyze” tab, the data were visible in a new window and saved as an Excel file. The values of IntDen from CCH‐treated tissues were further analyzed for fold change over control.

### 2.11 Statistical analysis

Rheometry data are presented as means ± SEM of CCH‐treated fibroids or untreated control fibroids. Statistical significance was determined using Wilcoxon matched‐pairs signed rank test using GraphPad Prism version 6.01 for Windows (GraphPad, San Diego, CA, USA). For proliferative cell nuclear antigen (PCNA) and p‐YAP analysis, data are presented as means of technical replicates (*n* = 5) from each CCH‐treated fibroid or each untreated control fibroid. We analyzed these data, using student's *t*‐test (two‐tailed and homoscedastic: two sample equal variance) in excel file. For in vitro data analysis, we used Mann Whitney U test to evaluate differences in fold change values between control and treatment group. A *p*‐value of <0.05 was considered statistically significant.

## 3 RESULTS

### 3.1 CCH injection regulates stiffness of fibroid tissues and cell proliferation as well as Hippo signaling

**FIGURE 1:**
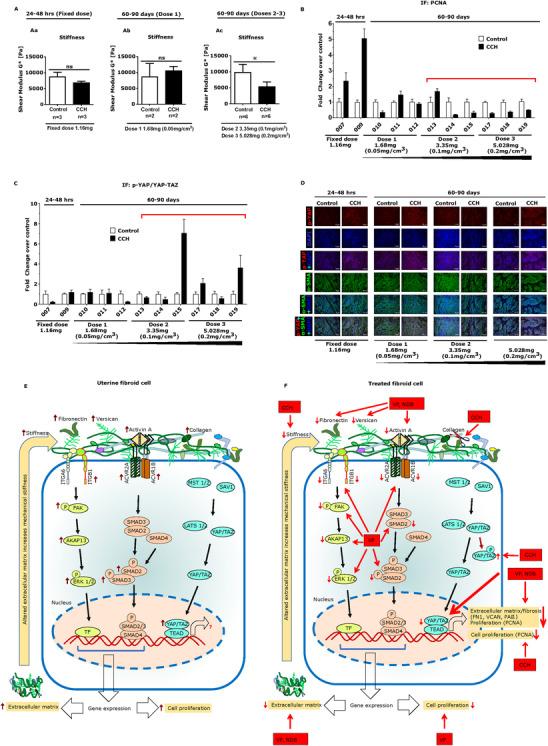
Collagenase *Clostridium histolyticum* (CCH) injection regulates stiffness of extracellular matrix and cell proliferation as well as Hippo signaling. Uterine fibroid tissues were injected with CCH at a fixed dose (1.16 mg) (*n* = 3), and tissues were collected after 24–48 h (Group 1). In group 2, fibroids were injected with CCH at 0.05 mg/cm^3^ (dose 1) (*n* = 3), 0.1 mg/cm^3^ (dose 2) (*n* = 3), and 0.2 mg/cm^3^ (dose 3) (*n* = 3) and were collected after 60–90 days. One sample was morcellated and was not able to be tested. Therefore, we excluded that sample from the analysis. (A) Quantification of mechanical stiffness in control and CCH‐treated fibroid tissues. Injection of CCH at fixed dose (1.16 mg) reduced stiffness by 21% (*p *= 0.500) (a). In contrast, CCH injection at low dose (0.05 mg/cm^3^) increased stiffness by 22% (*p *= 0.630) (b). Of note, stiffness of fibroid tissue was significantly reduced (46%) by CCH injection at the two highest doses (0.1–0.2 mg/cm^3^) (*p *= 0.031) (c). Data are presented as means ± SEM of CCH‐treated or control fibroids (these are considered as biological replicates). Statistical significance was determined using Wilcoxon matched‐pairs signed rank test and set a *p *< 0.05. *p < 0.05. (B) Levels of the proliferation marker, proliferative cell nuclear antigen (PCNA), in CCH‐treated fibroids. CCH injection increased PCNA expression in two out of two subjects in 24–48 h treatment group and decreased in five of six subjects in 60–90 days at highest doses (values below red bar) (*p *< 0.05). The numbers below the X‐bars (such as 007–019) indicate subject (or fibroid) number. (C) Quantification of the Hippo signaling factor phosphorylated YAP (*p*‐YAP) in control (open bars) or CCH‐treated fibroid samples (black bars). The levels of *p*‐YAP were normalized with YAP/TAZ (total). The *p*‐YAP expression was increased in 27% (three out of 11) CCH‐injected fibroids (black bars), compared to control samples (open bars). Notably, CCH at doses 2–3 increased *p*‐YAP expression in 50% (three out six) (values below red bar) CCH‐treated fibroid tissues (*p *< 0.05). Data are presented as mean ± SD of five images (these are considered as technical replicates) (20X) from CCH treated fibroid or control adjacent fibroid. (D) Representative immunofluorescence images of *p*‐YAP expression in control and CCH treated fibroids. Phospho‐YAP immunostaining is indicated as red, DAPI is indicated as blue, and α‐SMA is indicated as green. Scale bar = 50 μM. Negative controls were performed by omitting the first or the secondary antibody. (E) Schematic representation of mechanotransduction and growth factor signaling in fibroid growth. In fibroid cells, extracellular matrix (ECM) initiates mechanotransduction process by interacting with integrin receptors (ITGA6, ITGB1) and activates cytoplasmic kinase cascades (such as FAK, AKAP13). This leads to activate downstream signaling pathways (such as ERK 1/2) that regulates transcription of genes involved in cell growth and fibrosis. Profibrotic growth factor activin A binds to its receptors that activates SMAD2/3 signaling pathway and induces fibrotic gene expression. In fibroid tumors the Hippo target “YAP” is in the nucleus. In this state, Hippo signaling is OFF, the MST1/2 and LATS1/2 kinases are inactive, YAP and TAZ are dephosphorylated, and translocate into the nucleus where they are in complex with TEAD, inducing transcription of genes. (F) Regulation of multiple critical targets of fibroid growth by antifibrotic compounds through inactivation of YAP pathway. CCH‐treated fibroid shows a reduction in stiffness of ECM (46% reduction), reduction in staining intensity of PCNA (cell proliferation marker) (six out of 11 subjects) at high doses. There is an increase in phosphorylated YAP at high dose of CCH, suggesting the inactivation of YAP. The inhibitor of YAP, verteporfin inhibits series of targets of fibrosis and proliferation, at least in part, by inhibition of YAP, as well as ERK 1/2 and SMAD 2/3 signaling pathways. The antifibrotic drug, nintedanib also inhibits YAP in fibroid cells that leads to changes in the expression of genes involved in cell growth and fibrosis. These suggests that these three drugs might be used as alone or as a combination therapy for fibroid treatment

Uterine fibroids contain an excessive amount of collagen that contributes to increased tissue stiffness which is associated with activation of mechanotransduction processes that promote fibroid growth.^14^, ^15^, ^16^, ^17^ We hypothesized that the digestion of collagens would lead to changes in stiffness detectable by rheometry. Fibroids in premenopausal women who were scheduled for hysterectomy were injected with CCH at different doses or left untreated. Fibroids were collected at hysterectomy after 24–48 h or 60–90 days duration. Results of the rheometry analysis are shown in Figure 1A. Injection of CCH at fixed dose (1.16 mg) led to a reduction in stiffness by 21% (*p *= 0.500) (Figure 1Aa) compared to untreated control at 24–48 h. In contrast, CCH injection at low dose (0.05 mg/cm^3^) increased stiffness by 22% (*p *= 0.630) (Figure 1Ab) compared to control at 60–90 days. Notably, we observed a significant reduction (46%) in the stiffness of fibroid tissue samples at the two highest CCH doses (0.1–0.2 mg/cm^3^) at the 60–90 days (*p *= 0.031) (Figure 1Ac).

The findings suggested that CCH could induce significant reduction in stiffness of the ECM at higher dosages. We suspected that the softening of the fibroid tissues caused by CCH injection may change the growth phenotype of fibroid cells. In order to determine the effect of CCH on cell proliferation, we evaluated PCNA expression through immunofluorescence and ImageJ analysis. We found that PCNA expression was unexpectedly increased at the 24–48 h time‐point, after CCH injection with fixed dose (1.16 mg). However, as expected, PCNA expression was decreased in 83% (five out of six) fibroids after 60–90 days at the two higher doses (*p *< 0.05) (Figure 1B). This important finding suggests that injection with collagenase led to a reduced cell proliferation in the CCH‐injected fibroid tissues at 60–90 days which would favor regression of the fibroid tumors.

To explore the possible mechanisms whereby CCH effects on the ECM stiffness might influence cell proliferation, we focused on the Hippo pathway, a fundamental signaling cascade that integrates mechanical signaling with cell growth. YAP acts as a sensor and regulator of a wide range of physical and mechanical stress, including stiffness of ECM.^21^, ^22^, ^47^ The increased ECM stiffness activates YAP which promotes the transcription of cell growth and fibrotic genes.^19^, ^20^, ^21^ On the other hand, in soft matrix, YAP becomes phosphorylated or degraded in the cytoplasm.^19^, ^23^ When YAP is phosphorylated it becomes transcriptionally inactive. Based on the finding that CCH injection reduced stiffness of ECM, we therefore postulated that there may be higher levels of phospho‐YAP (p‐YAP) in CCH‐treated group. To quantify expression of p‐YAP, we stained fibroid tissues with p‐YAP antibody using immunofluorescence and measured staining intensities by ImageJ software. As shown in Figure 1C, 27% (three out of 11 samples) CCH‐treated samples exhibited an increase in p‐YAP following treatment with CCH. Notably, three out of six samples at the highest doses of CCH showed an increase in p‐YAP (Figure 1C) (*p *< 0.05), which would favor a reduction in fibroid growth. The representative images of increased p‐YAP staining after CCH treatment are shown in Figure 1D.

Overall, these results suggest that CCH‐treated uterine fibroids exhibited a reduction of ECM stiffness and cell proliferation, and inactivation of YAP (increased levels of phosphorylated YAP) at highest doses. To further explore the role of YAP in fibroid cell function, we next tested a small molecule inhibitor verteporfin (YAP inhibitor).^34^ We performed series of experiments to test the effect of verteporfin on gene and protein expression involved in cell proliferation, fibrosis, and mechanotransduction (important pathophysiological processes of fibroid growth) (Figure 1E) through in vitro cell culture model. We also introduced nintedanib to determine whether this anti‐fibrotic drug was effective in regulating major processes of fibroid growth, at least in part, by inactivation by YAP pathway. We observed (Figure 1F) the regulation of multiple critical targets of fibroid growth by antifibrotic compounds through inactivation of YAP pathway.

### 3.2 Verteporfin showed YAP‐specific actions in fibroid and myometrial cells

**FIGURE 2:**
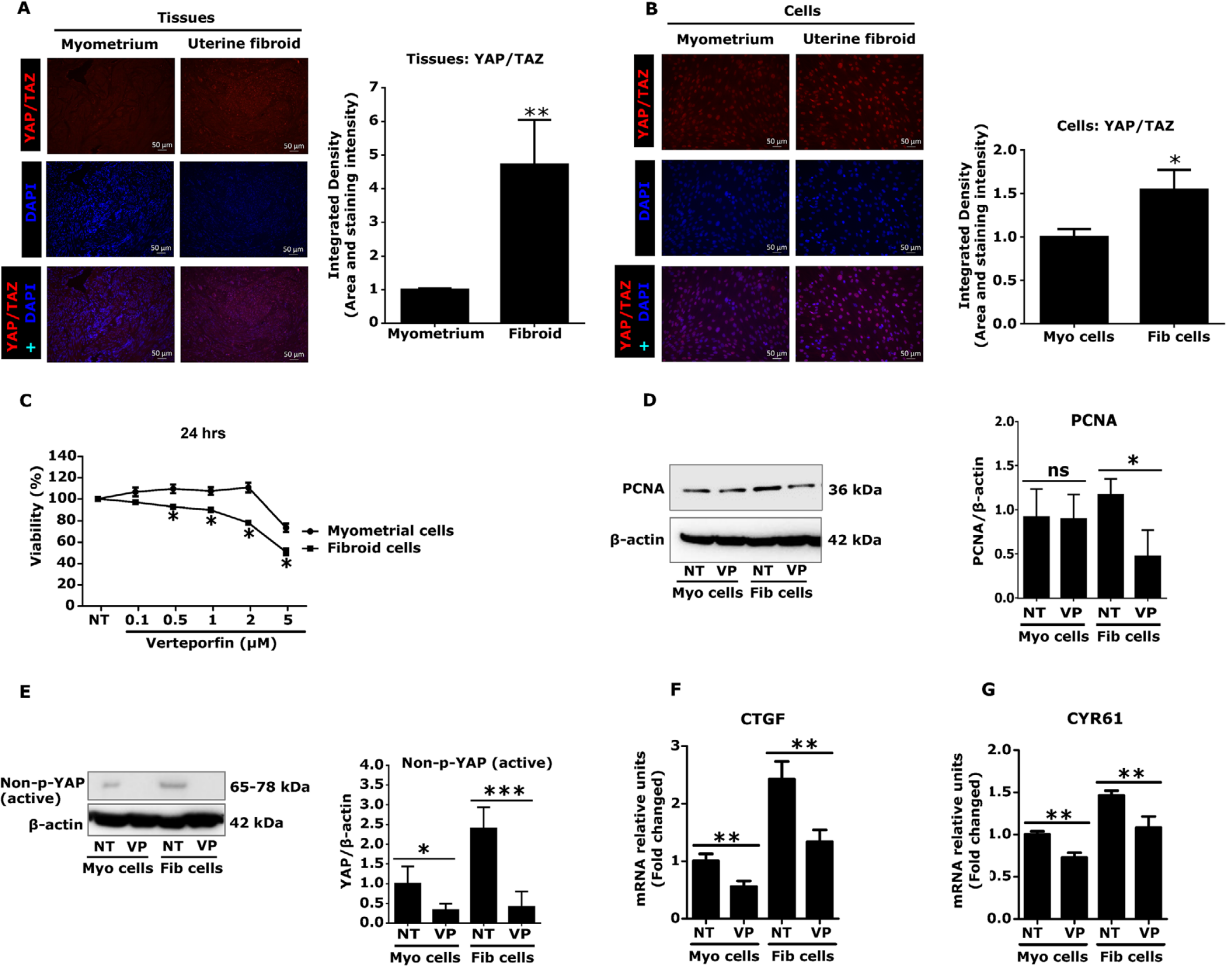
Verteporfin (VP) shows YAP‐specific actions in fibroid and myometrial cells. (A and B) The expression and localization of YAP/TAZ in myometrium and fibroid tissues. YAP/TAZ expression levels were quantified in myometrial and fibroid tissues (A) or cells (B). Both myometrium and fibroids expressed YAP/TAZ (nuclear) but intensity was higher in fibroids. Results are presented as mean ± SEM. For tissues, data were calculated from 10 immunofluorescence images of two patients. For cells, data were calculated from three independent experiments (*n* = 9) (20X). Scale bar = 50 μM. (C and D) VP reduced fibroid cell proliferation. MTS assays were performed after treatment with verteporfin for 24 h. The differential effect in the viability of fibroid cells was observed after VP treatment compared to myometrial cells (*n* = 4) (C). Results are presented as mean ± SEM. Data were calculated from four independent biologic experimental replicates. VP (1 μM) reduced protein (*n* = 5) expression of proliferative gene PCNA (proliferative cell nuclear antigen) in fibroid cells (D). (E‐G) YAP‐specific actions of VP in fibroid and myometrial cells. The reduced protein expression of non‐phospho‐YAP (active) in fibroid and myometrial cells after VP treatment (VP) for 24 h was compared to untreated (NT) control (*n* = 3) (E). The house keeping protein β‐actin was used to normalize non‐phospho‐YAP levels. The YAP‐responsive genes connective tissue growth factor (CTGF) (F) and CYR61 (cysteine‐rich angiogenic inducer 61) (G) were highly expressed in fibroid, compared to myometrial cells. VP significantly decreased mRNA levels in both fibroid and myometrial cells (*n* = 3). Each graph represents experimental data with means ± SD. Data were calculated from 3–5 independent biologic experimental replicates. Statistical significance was determined using the Mann Whitney U test and set at *p *< 0.05. *p < 0.05, **p < 0.01, ***p < 0.001. Abbreviation: NT, no treatment (control)

To confirm the actions of verteporfin on fibroid and myometrial cells, we first evaluated the expression and localization of YAP/TAZ in myometrium and fibroid tissues as well as in cells using immunofluorescence techniques. Data revealed that both myometrium and fibroid tissues expressed YAP/TAZ, but signal intensity was higher in fibroids (Figure 2A). We found a similar trend of YAP/TAZ expression in fibroid and myometrial cells (Figure 2B).

Having observed a differential expression of YAP/TAZ in fibroids and myometrium, we next tested viability of fibroid and myometrial cells treated with verteporfin. Human fibroid and myometrial cells were treated with verteporfin at different doses (0.1, 0.5, 1, 2, 5 μM) or left untreated (DMSO was used as a vehicle control) for 24 h. Results showed a dose dependent regulation of cell growth by verteporfin. Verteporfin at 1 and 2 μM inhibited fibroid cell growth by 10% and 22%, respectively, while myometrial cells were not affected (Figure 2C). The reduction in expression of protein (Figure 2D) levels of proliferative gene PCNA in fibroid cells was also evident. Based on data from the viability test, we selected 1 μM concentration of verteporfin for further experiments. In order to determine the effect of verteporfin on YAP and YAP‐responsive genes, fibroid and myometrial cells were treated with verteporfin for 24 h. The expression of protein and mRNA was measured through western blot and real time qPCR, respectively. We observed a clear reduction in protein expression of YAP (non‐p‐YAP; transcriptionally active) in both fibroid and myometrial cells (Figure 2E). The mRNA levels of YAP‐responsive genes connective tissue growth factor (CTGF) (2.4‐fold) (Figure 2F) and cysteine‐rich angiogenic inducer 61 (CYR61) (1.5‐fold) (Figure 2G) were found to be higher in fibroid, compared to myometrial cells. Verteporfin significantly inhibited transcript levels of CTGF (*p *< 0.01) (Figure 2F) and CYR61 (*p *< 0.01) (Figure 2G) in fibroid cells, further confirming its YAP‐specific actions. Furthermore, verteporfin reduced TEAD‐luc activity in fibroid cells, compared to untreated control cells (Figure S4). TEAD is a binding partner of YAP in the nucleus that regulates transcription of cell growth and fibrotic genes, such as CTGF and CYR61.

### 3.3 Verteporfin reduced key targets of fibrosis in fibroid cells

**FIGURE 3:**
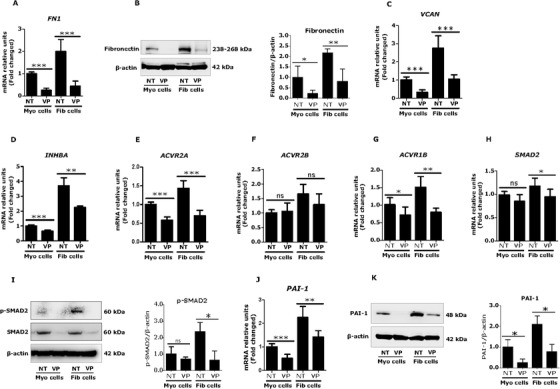
Verteporfin (VP) reduces key targets of fibrosis in fibroid cells. (A‐C) VP reduced extracellular matrix (ECM) expression in fibroid cells. Human fibroid and myometrial cells were treated with VP at 1 μM for 24 h. The mRNA and protein expression was measured using real time qPCR and western blot, respectively. (A and B) Fibronectin was highly expressed in fibroid compared to myometrial cells. VP decreased mRNA (*n* = 3) (A) and protein (*n* = 4) (B) levels in both fibroid and myometrial cells. (C) Versican (VCAN) was also highly expressed in fibroid compared to myometrial cells. VP significantly reduced mRNA expression in both fibroid and myometrial cells (*n* = 3) (C). (D‐K) VP reduced activin A, its receptors, and downstream targets in fibroid cells. Uterine fibroid cells highly expressed activin A (INHBA) (D) and its receptors, ACVR2A (E), ACVR2B (F), and ACVR1B (G) which were downregulated by VP treatment (*n* = 3). VP also decreased SMAD2 (H) and *p*‐SMAD2 (I) levels in fibroid cells (*n* = 3). The downstream target of activin A, PAI‐1 (plasminogen activator inhibitor‐1) mRNA (J) and protein (K) levels were reduced by verteporfin in fibroid and myometrial cells (*n* = 3). Results are expressed as mean ± SD. Data were calculated from 3–4 independent biologic experimental replicates. Statistical significance was determined using the Mann Whitney U test and set at *p *< 0.05. *p < 0.05, **p < 0.01, ***p < 0.001. Abbreviations: NT, no treatment (control)

Fibrosis was reported to be the most important cellular event in fibroid growth.^14^ This process is mediated by several ECM proteins and growth factor signaling.^14^, ^48^ To determine the effect of verteporfin on fibrosis‐related factors, we first measured expression levels of two proteins upregulated in fibroids, fibronectin and versican,^49^, ^50^ in response to verteporfin treatment in fibroid and myometrial cells. As expected, we observed upregulation of fibronectin in fibroid cells in both mRNA (2.0‐fold) (Figure 3A) and protein (2.2‐fold) levels (Figure 3B) compared to myometrial cells. Verteporfin significantly decreased both mRNA (*p *< 0.001) and protein (*p *< 0.01) levels of fibronectin in fibroid cells (Figures 3A and 3B). Similar to fibronectin, versican upregulation in fibroid cells was observed at mRNA (2.8‐fold) (Figure 3C) levels. Verteporfin greatly reduced this transcript (*p *< 0.001) levels in fibroid cells (Figure 3C). We also observed that verteporfin reduced levels of *COL1A1* transcripts in immortalized fibroid cells (Figure S5), but we did not detect changes in collagen protein (Figure S6) in the cells under the conditions tested.

Activin A is a member of TGF‐β superfamily which is overexpressed in fibroids^51^ and is known to increase the expression of ECM proteins (such as fibronectin and versican) and has been suggested to play a role in myofibroblastic transition in fibroid cells.^48^, ^52^, ^53^ In this study, we also found an increase in transcript levels of activin A (3.7‐fold) in fibroid compared to myometrial cells (Figure 3D). Verteporfin greatly reduced transcript levels of activin A (*p *< 0.01) (Figure 3D). The activin A receptors ACVR2A (1.4‐fold) (Figure 3E), ACVR2B (1.7‐fold) (Figure 3F), and ACVR1B (1.5‐fold) (Figure 3G) transcript levels were observed to be higher in fibroid compared to myometrial cells. We observed a significant decrease in levels of ACVR2A (*p *< 0.001) and ACVR1B (*p *< 0.01) but not ACVR2B (*p *= 0.15) by verteporfin treatment of fibroid cells (Figures 3E‐3G). Activin A is known to activate SMAD signaling in fibroid cells.^48^ Verteporfin inhibited SMAD2 mRNA levels in fibroid cells (*p *< 0.05) (Figure 3H). We also detected higher levels of phosphorylated SMAD2 (2.4‐fold) in fibroid, compared to myometrial cells, which was inhibited by verteporfin treatment (*p *< 0.05) (Figure 3I). PAI‐1 (plasminogen activator inhibitor‐1) is a downstream target of activin A and is known to mediate fibrosis.^54^ Data revealed that PAI‐1 was highly expressed at mRNA (2.3‐fold) (Figure 3J) and protein (2.1‐fold) (Figure 3K) levels in fibroid compared to myometrial cells, which was inhibited by verteporfin treatment (*p *< 0.05) (Figures 3J and 3K).

### 3.4 Verteporfin influences genes involved in mechanotransduction in fibroid cells

**FIGURE 4:**
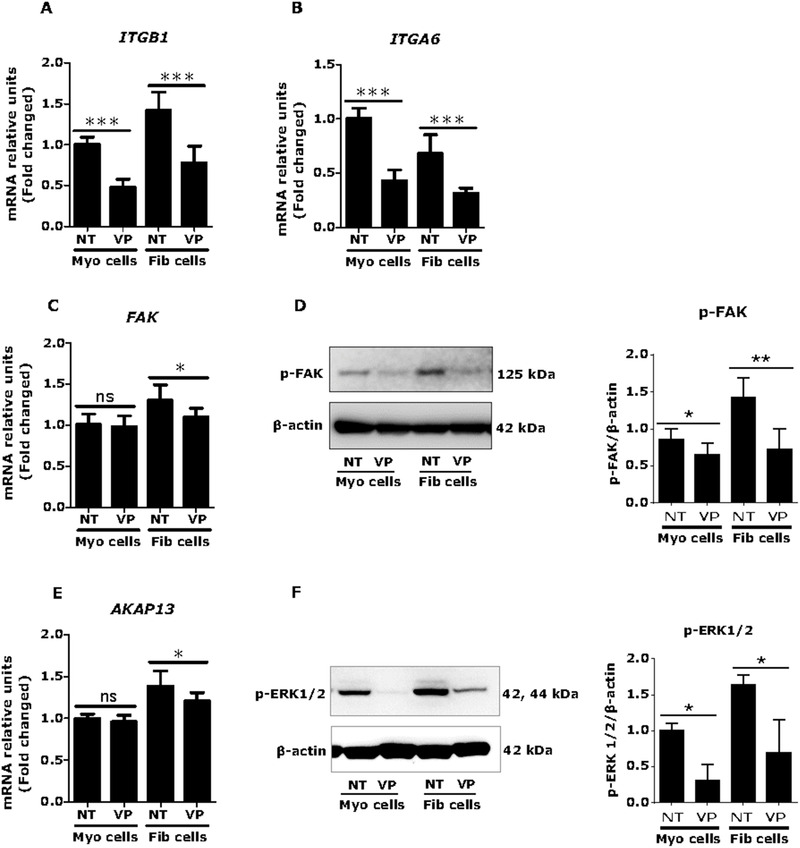
Verteporfin (VP) regulates of mechanotransduction in fibroid cells. Human fibroid and myometrial cells were treated with VP (1 μM) for 24 h. The mRNA and protein expressions of selected genes were measured through real time qPCR and western blot, respectively. The integrin receptors ITGB1 (A) but not ITGA6 (B) were more highly expressed in fibroid compared to myometrial cells and were reduced by VP treatment (*n* = 3). The cytoplasmic kinase focal adhesion kinase (FAK) (C) and phospho‐FAK (D) were also highly expressed in fibroid cells and were decreased by VP treatment (*n* = 3). A‐kinase anchor protein 13 (AKAP13) is a target of FAK that was expressed at higher levels in fibroid cells and likewise decreased by VP treatment (*n* = 3) (E). VP also reduced phospho‐ERK 1/2 (F) levels in both fibroid and myometrial cells (*n* = 3). Results are presented as mean ± SD. Data were calculated from three independent biologic experimental replicates. Statistical significance was determined using the Mann Whitney U test and set at *p *< 0.05. *p < 0.05, **p < 0.01, ***p < 0.001. Abbreviation: NT, no treatment (control)

Mechanotransduction is a process by which cells translate mechanical stress into biochemical signals.^55^ The ECM affects cell function through physical interaction with integrin receptors (ITGA6 and ITGB1) and activates cytoplasmic kinase cascades following activation of downstream signaling pathways such as MAPK (mitogen‐activated protein kinase). This process leads to increase gene expression involved in cell growth and fibrosis. We found that integrin receptors ITGB1 (1.4‐fold) (Figure 4A) but not ITGA6 (0.7‐fold) (Figure 4B) were highly expressed in fibroid compared to myometrial cells. Verteporfin decreased those transcript levels in both fibroid (*p *< 0.001) and myometrial cells (*p *< 0.001) (Figures 4A and 4B). Focal adhesion kinase (FAK) is a cytoplasmic kinase that elevated (mRNA) (1.3‐fold) in fibroid compared to myometrial cells which was downregulated by verteporfin treatment (*p *< 0.05) (Figure 4C). The protein levels of phosphorylated FAK were also increased (1.5‐fold) in fibroid cells and verteporfin reduced the levels (*p *< 0.01) (Figure 4D). A‐kinase anchoring protein 13 (AKAP13), a target of FAK, was found to be increased (mRNA) (1.4‐fold) in fibroid cells which was suppressed by verteporfin treatment (*p *< 0.01) (Figure 4E). Furthermore, we found increased levels of phosphorylated ERK1/2 (1.6‐fold) in fibroid compared to myometrial cells, which was inhibited by verteporfin treatment (*p *< 0.05) (Figure 4F). These results suggest that verteporfin efficiently targeted upstream and downstream of mechanotransduction process in fibroid cells.

We interpret the data in Figures 2, 3, 4 to indicate that inhibition of YAP via verteporfin may reduce fibroid cell growth and expression of key targets of fibrosis and mechanotransduction associated with downregulation of SMAD and ERK signaling pathways. Next, we introduced the antifibrotic drug nintedanib as another approach to validate our target YAP as promoters of key pathophysiological processes involved in fibroid growth.

### 3.5 Nintedanib treatment reduced growth of fibroid cells and expression of genes or proteins involved in fibrosis, at least in part, by alteration of Hippo pathway

**FIGURE 5:**
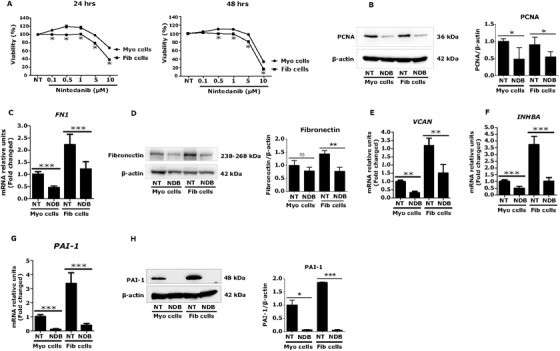
Nintedanib (NDB) treatment reduces growth of fibroid cells and expression of genes or proteins involved in fibrosis. (A and B) Nintedanib treatment reduced proliferation of fibroid cells. Human fibroid and myometrial cells were treated with NDB (5 μM) for 24 h and 48 h or cells were treated with vehicle. An MTS assay was performed after treatment with NDB for indicated durations. The differential effect of NDB was observed in cell proliferation of fibroid cells versus myometrial cells (*n* = 4) (A). Results are presented as mean ± SEM. Data were calculated from four independent biologic experiments. NDB reduced PCNA protein expression in both myometrial and fibroid cells, compared to untreated controls (*n* = 3) (B). (C‐E) NDB treatment decreased expression of key targets of fibrosis in fibroid cells. Myometrial and fibroid cells were treated with NDB at 5 μM concentration for 24 h. The mRNA and protein expression of selected genes was measured using real time qPCR and western blot, respectively. Fibronectin (FN) was highly expressed at both mRNA (C) and protein (D) levels in fibroid than myometrial cells, and levels were reduced by NDB treatment (*n* = 3). Versican (VCAN) was also highly expressed in fibroid cells and levels were reduced by NDB treatment (*n* = 2) (E). (F‐H) NDB treatment decreased activin A and its downstream target in fibroid cells. Activin A (INHBA) was highly expressed in fibroid cells, compared to myometrial cells, and levels were reduced by nintedanib treatment (*n* = 3) (F). The mRNA and protein levels of PAI‐1 (plasminogen activator inhibitor‐1) were higher in fibroid, compared to myometrial cells. NDB treatment decreased PAI‐1 mRNA (*n* = 3) (G) and protein (*n* = 2) (H) levels in both fibroid and myometrial cells. Results are presented as mean ± SD. Data were calculated from 2–4 independent biologic experiments. Statistical significance was determined using the Mann Whitney U test and set at *p *< 0.05. *p < 0.05, **p < 0.01, ***p < 0.001. Abbreviation: NT, no treatment (vehicle)

To evaluate the effect of nintedanib on cell proliferation, human fibroid and myometrial cells were treated with nintedanib at different concentrations (0.1, 0.5, 1, 5, and 10 μM) for 24 or 48 h. We found that myometrial and fibroid cells proliferated at lower concentrations (0.1–1 μM) of nintedanib, while a differential effect of nintedanib treatment was observed at 5 μM for 24 or 48 h, with a ≥20% reduction of fibroid cell growth, and no effect on myometrial cells (Figure 5A). The protein levels of the proliferation marker, PCNA were decreased after 48 h of nintedanib treatment in both cell types (*p *< 0.05) (Figure 5B).

Next, we evaluated the effect of nintedanib on fibrosis‐related factors. Human myometrial and leiomyoma cells were treated with nintedanib at 5 μM for 24 h, and mRNA and protein levels of fibrosis‐related molecules (fibronectin and versican) were measured by real time qPCR and western blot, respectively. These two ECM proteins are reported to be overexpressed in uterine fibroids.^49^, ^50^ As expected, mRNA (2.2‐fold) (Figure 5C) and protein (1.4‐fold) (Figure 5D) levels of fibronectin were higher in fibroid, compared to myometrial cells. Nintedanib greatly reduced both mRNA (*p *< 0.01) and protein (*p *< 0.01) levels of fibronectin in fibroid cells (Figures 5C and 5D). In addition to fibronectin, versican transcript levels were found to be higher (3.2‐fold) in fibroid compared to myometrial cells (Figure 5E). Nintedanib robustly decreased versican mRNA levels in fibroid (and myometrial cells (*p *< 0.01) (Figure 5E).

TGF‐β superfamily members, such as activin A are over expressed in fibroids and are known to increase ECM proteins^48^; therefore, we aimed to determine if nintedanib could reduce activin A and its downstream target, PAI‐1, expression in fibroid and myometrial cells. As expected, activin A (INHBA) was found to be higher (3.7‐fold) in fibroid cells at baseline and nintedanib treatment significantly reduced mRNA levels of INHBA (*p *< 0.001) (Figure 5F). PAI‐1 appears to play an important role in the progression to fibrosis,^56^ and its expression is induced by activin A in fibroid cells.^57^ We found that PAI‐1 was highly expressed at both mRNA (3.4‐fold) and protein (1.9‐fold) levels in fibroid, compared to myometrial cells (Figures 5G and 5H). Nintedanib treatment greatly reduced both mRNA (Figure 5G) (*p *< 0.001) and protein (Figure 5H) (*p *< 0.001) levels of PAI‐1 in fibroid cells.

**FIGURE 6:**
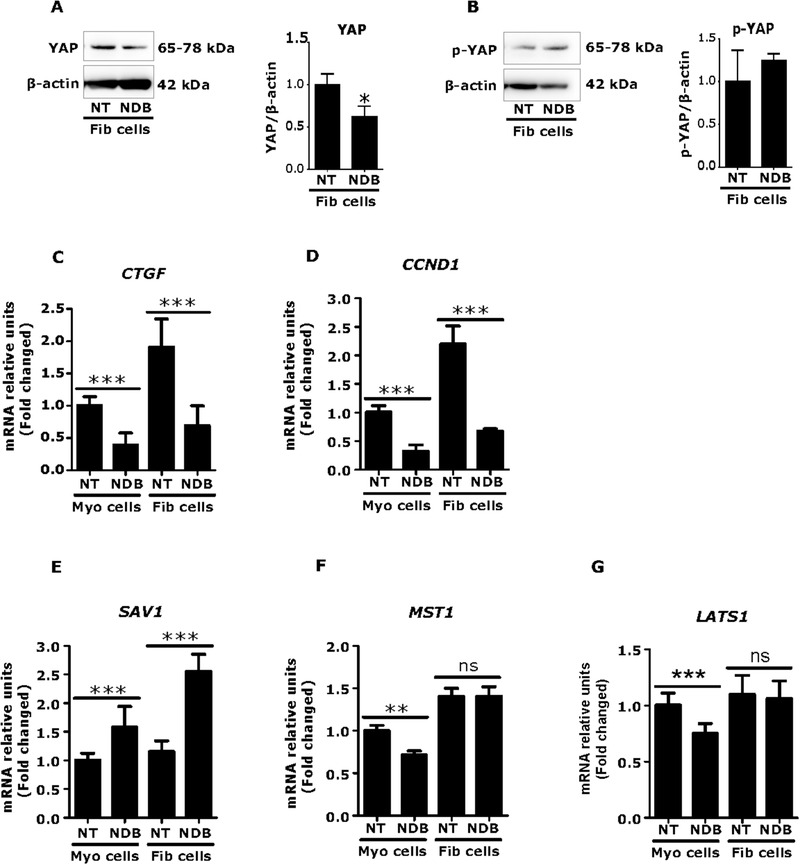
Nintedanib (NDB) treatment alters Hippo pathway in fibroid cells. Human fibroid and myometrial cells were treated with NDB (5 μM) for 24 h. NDB treatment decreased yes‐associated protein (YAP ) (A) and slightly increased phospho‐YAP (B) in fibroid cells (*n* = 3). The house keeping protein β‐actin was used to normalize YAP and phospho‐YAP levels. The YAP‐responsive genes connective tissue growth factor (CTGF) (C) and CCND1 (cyclin D1) (D) levels were reduced by NDB treatment of fibroid and myometrial cells (*n* = 3). The Hippo kinases SAV1 (Salvador Family WW Domain Containing Protein 1) (E) was increased but MST1 (macrophage stimulating 1) (F), and LATS1 (large tumor suppressor 1) (G) levels were unchanged after NDB treatment (*n* = 3). Results are expressed as mean ± SD. All the data were calculated from three independent biologic experiments. Statistical significance was determined using the Mann Whitney U test and set at *p *< 0.05. *p < 0.05, **p < 0.01, ***p < 0.001. Abbreviation: NT, no treatment (vehicle)

Having observed the anti‐fibrotic effects of nintedanib in fibroid cells, we next investigated whether nintedanib could influence YAP and YAP‐responsive gene expression. Data showed that the protein levels of Hippo pathway effector YAP were significantly reduced after 24 h of nintedanib treatment in fibroid cells (*p *< 0.05) (Figure 6A). As expected, p‐YAP protein expression levels were slightly increased by nintedanib treatment (1.24‐fold) (*p *= 0.32) (Figure 6B), suggesting the inactivation of YAP in cytoplasm. Accordingly, YAP‐responsive genes, CTGF (*p *< 0.001) (Figure 6C) and CCND1 (*p *< 0.001) (Figure 6D), were decreased by nintedanib treatment in fibroid cells. Furthermore, mRNA levels of the Hippo pathway kinases SAV1 were significantly increased (2.6‐fold) (*p *< 0.001), while MST1 (*p *= 1.00) and LATS1 (*p *= 0.62) were not affected by nintedanib treatment in fibroid cells, compared to untreated controls (Figures 6E‐6G). Taken together, the results suggest that the anti‐proliferative and anti‐fibrotic effects of nintedanib are mediated, at least in part, through inactivation of YAP.

## 4 DISCUSSION

Based on the crucial role of fibrosis in fibroid growth, we postulated that collagenase injection of uterine fibroids would be accompanied by an altered growth phenotype of resident cells which might be accompanied by changes in the Hippo signaling pathway. Although this study had a small size, the results demonstrated that injection of fibroids with collagenase was associated with reduction in mechanical stiffness and reduced cell proliferation at higher CCH doses, which was accompanied by elevated levels of phosphorylated YAP. As this was a phase 1/2A study, future studies will be required to determine the dosage and clinical outcomes following CCH injection. We also assessed levels of collagen in the treated fibroid samples and observed that CCH significantly reduced collagen content at all doses (Figure S7). There was variability between subjects, likely related to the heterogeneity of uterine fibroids and differing levels of collagen. Digestion of collagen appeared to be too sensitive a marker to assess drug effects, but rheometry indicated a clear dose‐dependent response accompanied by changes in PCNA and Hippo signaling factors. It should be noted that fibroids are heterogenous tumors, and the observation that all fibroids studied showed variable findings following injection of collagenase is likely explained by the heterogeneity of the condition and only strengthens the findings reported in this paper.^58^ Furthermore, inhibition of YAP via verteporfin and nintedanib was associated with reduction of fibroid cell proliferation, as well as reduction of genes or proteins involved in fibrosis and mechanotransduction that are important pathophysiological events of uterine fibroid growth. These results suggested that these drugs might be effective in regulating fibroid growth by alteration of the Hippo pathway (Figure 1F).

We observed an increased level of p‐YAP in three of six fibroids at the high doses of CCH compared to control tissue. The increase in phosphorylated YAP is consistent with reduction in Hippo target gene expression and reduced cell growth. We observed dose‐dependent changes in the cell proliferation marker, PCNA, with reduction in cell proliferation at the higher doses for 60–90 days. This notable finding suggests that over time collagenase treatment might lead to a reduction in fibroid size. We also tested the effect of CCH on apoptosis by TUNEL assay but did not observe an increase in apoptosis, contrary to our expectations. Interestingly, results showed that CCH treatment induced cell death in some CCH‐treated fibroids through autophagy. For autophagy, we used LC3B, one of the proteins involved in the formation of autophagosomes. We measured and analyzed LC3B expression through immunofluorescence and ImageJ software (Figure S8). LC3B levels were increased in 55% (six of 11) CCH‐treated fibroids.

To explore the role of YAP in fibroid growth, we introduced an inhibitor of YAP function, verteporfin.^34^ YAP overexpression is associated with fibrosis of many organs such as lung,^59^ kidney,^60^ and liver.^61^ Fibrosis is one of the main characteristics of uterine fibroids.^14^ Not surprisingly, YAP overexpression is observed in uterine fibroids.^62^ In this study, we also found a similar trend of YAP expression in uterine fibroids. Next, we demonstrated the effect of verteporfin on multiple targets of the Hippo pathway, cell growth, fibrosis, and mechanotransduction in uterine fibroid cells. As expected, we found a clear reduction of YAP in both fibroid and myometrial cells after verteporfin treatment that confirms its YAP‐specific actions. CTGF, a known direct TAZ‐TEAD target gene (TAZ is a paralogue of YAP that interacts in the nucleus with TEAD), is one of the main players in fibrosis.^63^ Verteporfin treatment greatly reduced CTGF expression in fibroid cells. Additionally, the Hippo downstream effector, CYR61 (CCN1) is a known mediator of cell proliferation and migration.^64^ CYR61 was overexpressed in fibroid compared to myometrial cells, which was attenuated by verteporfin treatment. These data further confirm the YAP‐specific actions of verteporfin.

The central components of fibrosis are ECM proteins. One of the proteins is fibronectin, a large glycoprotein of ECM that serves as a general cell adhesion molecule by anchoring cells to a variety of ECM types. The ED‐A‐containing polymerized fibronectin is crucial for the induction of the myofibroblastic phenotype, a cell responsible for release of cytokines, growth factors, and accumulation of ECM proteins, a main phenomenon of fibrotic process.^65^ Versican is a large ECM proteoglycan that plays important role in cell‐matrix interactions and is involved in inflammatory and fibrotic disorders.^66^, ^67^ Both ECM components fibronectin and versican are overexpressed in fibroids.^49^, ^50^ In this study, we noticed overexpression of fibronectin and versican in fibroid cells which was robustly inhibited by verteporfin treatment. These results suggest a potential treatment of fibroids due to the antifibrotic effect of verteporfin.

As expected, we found higher levels of profibrotic growth factor, activin A in fibroid cells, which was significantly reduced by verteporfin treatment. Importantly, verteporfin treatment was associated with reduction in expression of activin receptors (ACVR2A and ACVR1B) and cytoplasmic kinases (SMAD2) and active transcriptional effector (phosphorylated SMAD2). PAI‐1 is a downstream target of activin A mediated signaling which is a known mediator of fibrosis.^54^ We found that PAI‐1 was highly expressed in fibroid cells and was reduced by verteporfin treatment. These results indicate that effects of verteporfin on the ECM in fibroid cells are partly mediated by downregulation of the activin A mediated SMAD signaling pathway.

In this study, we found that integrin receptors ITGB1, but not ITGA6, were highly expressed in fibroid cells, and both were decreased by verteporfin treatment. Verteporfin also decreased expression of FAK (phosphorylated) and AKAP13 levels. Importantly, verteporfin reduced downstream target ERK 1/2 (phosphorylated) levels in fibroid cells. This might be one of the explanations of inhibitory effect of verteporfin on cell growth and fibrosis in fibroid cells. We recently demonstrated that the lipid‐lowering drug simvastatin reduced ECM production and corrected the altered state of mechanotransduction in fibroids.^45^, ^68^ In addition, available literature shows that statins can prevent YAP‐mediated transcriptional activation of RHAMM (receptor for hyaluronan‐mediated motility).^69^ Therefore, it may be intriguing to investigate potentially synergistic combination therapy targeting ECM stiffness, mechanotransduction and YAP signaling in fibroids.

The above data indicate that YAP is highly expressed in fibroids. Inhibition of YAP via verteporfin decreased cell growth and gene or protein expression of multiple targets of fibrosis and mechanotransduction. These findings were associated with downregulation of SMAD and ERK1/2 pathways. These results suggest a potential role of YAP in fibroid growth. For further clarification of YAP's role in fibroid growth, we introduced the antifibrotic drug, nintedanib. Nintedanib is an FDA‐ approved drug for the treatment of idiopathic pulmonary fibrosis, and for the systemic sclerosis‐associated interstitial lung disease (SSc‐ILD). We hypothesized that nintedanib might exert antifibrotic effects in fibroid cells through inactivation of YAP. The expression levels of fibrosis‐related components (FN1, VCAN, INHBA, and PAI‐1) were greatly reduced by nintedanib treatment in fibroid cells. Interestingly, we found that the Hippo pathway was altered by nintedanib treatment; YAP protein levels were reduced while SAV1 mRNA and phospho‐YAP protein were increased, suggesting the inactivation of YAP in cytoplasm. Indeed, expression of the YAP‐responsive genes, CTGF and CCND1, was decreased by nintedanib treatment in fibroid cells. These new observations suggest that anti‐proliferative and anti‐fibrotic effects of nintedanib in fibroid cells are mediated, at least in part, by inactivation of YAP pathway.

This is the first *in vivo* study to characterize the cellular effects and mechanisms of action of collagenase on fibrotic processes in uterine fibroids. The main limitation is of this study is that the sample size included fibroids from only 12 patients and, as expected, we observed variable effects among the samples. Although variable, the results were consistent at high doses. The important observation is that CCH effect may be mediated by alteration of Hippo signaling. To address this limitation, the effective dose and duration of the treatment as well as the larger sample size are necessary to optimize CCH as a clinically useful treatment for fibroids. In future studies, we plan to quantify protein and gene expression studies in CCH‐treated tissues, but we were unable to perform the studies currently due to insufficient samples. In the present study, we explored the role of Hippo signaling in fibroid growth by the utilization of two inhibitors, verteporfin (YAP inhibitor), and nintedanib (RTK inhibitor). Interestingly, we observed differential effects of verteporfin and nintedanib on cell viability, as well as gene and protein expression between fibroid tumors and normal myometrial cells. These observations suggest that these two drugs might be effective against pathological cells without affecting normal cells in human tissues.

In conclusion, the data presented here indicate that targeting the Hippo pathway (via inactivation of YAP) by collagenase was associated with reduction of ECM stiffness and cell proliferation (Figure 1F). Functionally, the inactivation of YAP via verteporfin and nintedanib was associated with decreased cell proliferation, as well as reduced expression of genes or proteins involved in fibrosis and mechanotransduction (Figure 1F). The potential effects of CCH, verteporfin, and nintedanib against these active processes of fibroid growth further support their validation in larger studies. Additionally, combination treatments might be especially effective, since collagenase would digest and degrade collagens at the extracellular level, while verteporfin and nintedanib might inhibit the production of ECM and critical targets of fibroid growth at transcriptional and translational levels.

## CONFLICT OF INTEREST

James H. Segars is or was a PI on research sponsored by Bayer, Abbvie, Biospecifics, Allergan, Inc., and Myovant. James H. Segars serves on boards of the Society for Reproductive Investigation, and the American Gynecological and Obstetrical Society. Md Soriful Islam, Friederike L. Jayes, Bhuchitra Singh, Sadia Afrin, Joshua T. Brennan, and Phyllis C. Leppert have nothing to disclose. Mostafa A. Borahay serves as Advisory Board member for Myovant Sciences.

## AUTHOR CONTRIBUTIONS

*Study concept and design*: James H. Segars and Md Soriful Islam. *Methodology‐immunofluorescence staining and capture images of collagenase treated fibroid tissues*: Md Soriful Islam, Sadia Afrin, Bhuchitra Singh, and Joshua T. Brennan. *Methodology‐rheometry*: Friederike L. Jayes. *Methodology‐in vitro cell culture experiments*: Md Soriful Islam. *Analysis and interpretation of data*: Md Soriful Islam, Sadia Afrin, Bhuchitra Singh, Friederike L. Jayes, and Joshua T. Brennan. *Drafting of the manuscript*: Md Soriful Islam and James H. Segars. *Critical revision of the manuscript for important intellectual content*: Sadia Afrin, Friederike L. Jayes, Joshua T. Brennan, Mostafa A. Borahay, Phyllis C. Leppert, and James H. Segars. *Approval of the final manuscript*: Md Soriful Islam, Sadia Afrin, Bhuchitra Singh, Friederike L. Jayes, Joshua T. Brennan, Mostafa A. Borahay, Phyllis C. Leppert, and James H. Segars.

## DATA AND MATERIALS AVAILABILITY

All data associated with this study are present in the paper or the Supplementary Materials. The data that support the findings of this study are available from the corresponding author upon reasonable request.

## DISCLOSURE STATEMENT

None of the authors have financial relationships directly relevant to the manuscript. James H. Segars reports past, or present, research funding for clinical trials from BioSpecifics, Inc., Bayer, Abbvie and Obseva. James H. Segars has served as a consultant to Bayer and Myovant. James H. Segars serves on the following boards: Society for Reproductive Investigation, American Gynecological and Obstetrical Society.

## Supporting information

SUPPORTING INFORMATION

SUPPORTING INFORMATION

SUPPORTING INFORMATION

SUPPORTING INFORMATION

SUPPORTING INFORMATION

SUPPORTING INFORMATION

SUPPORTING INFORMATION

SUPPORTING INFORMATION

SUPPORTING INFORMATION

SUPPORTING INFORMATION

SUPPORTING INFORMATION

## References

[1] Day Baird D , Dunson DB , Hill MC , Cousins D , Schectman JM . High cumulative incidence of uterine leiomyoma in black and white women: ultrasound evidence. Am J Obstet Gynecol. 2003;188(1):100–107. 12548202 10.1067/mob.2003.99

[2] Buttram Jr VC , Reiter RC . Uterine leiomyomata: etiology, symptomatology, and management. Fertil Steril. 1981;36(4):433–445. 7026295 10.1016/s0015-0282(16)45789-4

[3] Go V , Thomas M , Singh B , et al. A systematic review of the psychosocial impact of fibroids before and after treatment. Am J Obstet Gynecol. 2020;223(5):674–708.e678. 32474012 10.1016/j.ajog.2020.05.044

[4] Islam MS , Protic O , Toti P , et al. Uterine leiomyoma: available medical treatments and new possible therapeutic options. J Clin Endocrinol Metab. 2013;98(3):921–934. 23393173 10.1210/jc.2012-3237

[5] Taylor DK , Leppert PC . Treatment for uterine fibroids: searching for effective drug therapies. Drug Discov Today Ther Strateg. 2012;9(1):e41. 23264802 10.1016/j.ddstr.2012.06.001 PMC3525705

[6] Friedman AJ , Hoffman DI , Comite F , Browneller RW , Miller JD . Treatment of leiomyomata uteri with leuprolide acetate depot: a double‐blind, placebo‐controlled, multicenter study. The Leuprolide Study Group. Obstet Gynecol. 1991;77(5):720–725. 1901638

[7] Schlaff WD , Ackerman RT , Al‐Hendy A , et al. Elagolix for heavy menstrual bleeding in women with uterine fibroids. N Engl J Med. 2020;382(4):328–340. 31971678 10.1056/NEJMoa1904351

[8] Donnez J , Tatarchuk TF , Bouchard P , et al. Ulipristal acetate versus placebo for fibroid treatment before surgery. N Engl J Med. 2012;366(5):409–420. 22296075 10.1056/NEJMoa1103182

[9] Leather AT , Studd JWW , Watson NR , Holland EFN . The prevention of bone loss in young women treated with GnRH analogues with “add‐back” estrogen therapy. Obstet Gynecol. 1993;81(1):104–107. 8416441

[10] Donnez J . Liver injury and ulipristal acetate: an overstated tragedy?. Fertil Steril. 2018;110(4):593–595. 30196943 10.1016/j.fertnstert.2018.06.044

[11] Cardozo ER , Clark AD , Banks NK , Henne MB , Stegmann BJ , Segars JH . The estimated annual cost of uterine leiomyomata in the United States. Am J Obstet Gynecol. 2012;206(3):211. 22244472 10.1016/j.ajog.2011.12.002 PMC3292655

[12] Mäkinen N , Mehine M , Tolvanen J , et al. MED12, the mediator complex subunit 12 gene, is mutated at high frequency in uterine leiomyomas. Science. 2011;334(6053):252–255. 21868628 10.1126/science.1208930

[13] Yang Q , Mas A , Diamond MP , Al‐Hendy A . The mechanism and function of epigenetics in uterine leiomyoma development. Reprod Sci. 2016;23(2):163–175. 25922306 10.1177/1933719115584449 PMC5933172

[14] Islam MS , Ciavattini A , Petraglia F , Castellucci M , Ciarmela P . Extracellular matrix in uterine leiomyoma pathogenesis: a potential target for future therapeutics. Hum Reprod Update. 2018;24(1):59–85. 29186429 10.1093/humupd/dmx032

[15] Leppert PC , Baginski T , Prupas C , Catherino WH , Pletcher S , Segars JH . Comparative ultrastructure of collagen fibrils in uterine leiomyomas and normal myometrium. Fertil Steril. 2004;82(Suppl 3):1182–1187. 15474093 10.1016/j.fertnstert.2004.04.030 PMC4137471

[16] Rogers R , Norian J , Malik M , et al. Mechanical homeostasis is altered in uterine leiomyoma. Am J Obstet Gynecol. 2008;198(4):474. e471–474. e411. 18395046 10.1016/j.ajog.2007.11.057 PMC2696475

[17] Norian JM , Owen CM , Taboas J , et al. Characterization of tissue biomechanics and mechanical signaling in uterine leiomyoma. Matrix Biol. 2012;31(1):57–65. 21983114 10.1016/j.matbio.2011.09.001 PMC3576015

[18] Malik M , Norian J , McCarthy‐Keith D , Britten J , Catherino WH . Why leiomyomas are called fibroids: the central role of extracellular matrix in symptomatic women. Semin Reprod Med. 2010;28(3):169–179. 20414841 10.1055/s-0030-1251475

[19] Pocaterra A , Romani P , Dupont S . YAP/TAZ functions and their regulation at a glance. J Cell Sci. 2020;133(2):jcs230425. 31996398 10.1242/jcs.230425

[20] Meng Z , Moroishi T , Guan K‐L . Mechanisms of Hippo pathway regulation. Genes Dev. 2016;30(1):1–17. 26728553 10.1101/gad.274027.115 PMC4701972

[21] Dupont S , Morsut L , Aragona M , et al. Role of YAP/TAZ in mechanotransduction. Nature. 2011;474(7350):179. 21654799 10.1038/nature10137

[22] Lampi MC , Reinhart‐King CA . Targeting extracellular matrix stiffness to attenuate disease: from molecular mechanisms to clinical trials. Sci Transl Med. 2018;10(422):eaao0475. 29298864 10.1126/scitranslmed.aao0475

[23] Meng Z , Qiu Y , Lin KC , et al. RAP2 mediates mechanoresponses of the Hippo pathway. Nature. 2018;560(7720):655–660. 30135582 10.1038/s41586-018-0444-0 PMC6128698

[24] Noguchi S , Saito A , Nagase T . YAP/TAZ signaling as a molecular link between fibrosis and cancer. Int J Mol Sci. 2018;19(11):3674. 10.3390/ijms19113674 PMC6274979 30463366

[25] Islam MS , Maher JY , Afrin S , Su S‐C , Segars J . Verteporfin inhibits fibrosis, inflammation and angiogenesis related genes in uterine fibroid cells. Fertil Steril. 2019;112(3):e349.

[26] Purdy MP , Ducharme M , Haak AJ , et al. YAP/TAZ are activated by mechanical and hormonal stimuli in myometrium and exhibit increased baseline activation in uterine fibroids. Reprod Sci. 2020;27(4):1074–1085. 32056132 10.1007/s43032-019-00106-4

[27] Desai SS , Hentz VR . Collagenase clostridium histolyticum for Dupuytren's contracture. Expert Opin Biol Ther. 2010;10(9):1395–1404. 20666587 10.1517/14712598.2010.510509

[28] Tsambarlis PN , Yong R , Levine LA . Limited success with clostridium collagenase histolyticum following FDA approval for the treatment of Peyronie's disease. Int J Impot Res. 2019;31(1):15. 30120383 10.1038/s41443-018-0063-1

[29] Levine LA , Schmid TM , Emeigh Hart SG , Tittelbach T , McLane MP , Tursi JP . Collagenase Clostridium histolyticum degrades type I and III collagen while sparing type iv collagen in vitro in Peyronie's plaque explants. J Urol. 2014;191(4S):e672–e673.

[30] Stewart EA , Friedman AJ , Peck K , Nowak RA . Relative overexpression of collagen type I and collagen type III messenger ribonucleic acids by uterine leiomyomas during the proliferative phase of the menstrual cycle. J Clin Endocrinol Metab. 1994;79(3):900–906. 8077380 10.1210/jcem.79.3.8077380

[31] Jayes FL , Liu B , Moutos FT , Kuchibhatla M , Guilak F , Leppert PC . Loss of stiffness in collagen‐rich uterine fibroids after digestion with purified collagenase Clostridium histolyticum. Am J Obstet Gynecol. 2016;215(5):596. e591–596. e598. 27177523 10.1016/j.ajog.2016.05.006

[32] Brunengraber LN , Jayes FL , Leppert PC . Injectable Clostridium histolyticum collagenase as a potential treatment for uterine fibroids. Reprod Sci. 2014;21(12):1452–1459. 25332216 10.1177/1933719114553449 PMC4231132

[33] Bierbaumer L , Katschnig AM , Radic‐Sarikas B , et al. YAP/TAZ inhibition reduces metastatic potential of Ewing sarcoma cells. Oncogenesis. 2021;10(1):1–13. 33419969 10.1038/s41389-020-00294-8 PMC7794350

[34] Liu‐Chittenden Y , Huang B , Shim JS , et al. Genetic and pharmacological disruption of the TEAD–YAP complex suppresses the oncogenic activity of YAP. Genes Dev. 2012;26(12):1300–1305. 22677547 10.1101/gad.192856.112 PMC3387657

[35] Mohede DC , de Jong IJ , Bank RA , van Driel MF . Verteporfin as a Medical Treatment in Peyronie's Disease. Sex Med. 2018;6(4):302–308. 30274909 10.1016/j.esxm.2018.08.002 PMC6302152

[36] Li J , Yao M , Zhu X , et al. YAP‐induced endothelial‐mesenchymal transition in oral submucous fibrosis. J Dent Res. 2019;98(8):920–929. 31282845 10.1177/0022034519851804

[37] Richeldi L , Du Bois RM , Raghu G , et al. Efficacy and safety of nintedanib in idiopathic pulmonary fibrosis. N Engl J Med. 2014;370(22):2071–2082. 24836310 10.1056/NEJMoa1402584

[38] Distler O , Highland KB , Gahlemann M , et al. Nintedanib for systemic sclerosis–associated interstitial lung disease. N Engl J Med. 2019;380(26):2518–2528. 31112379 10.1056/NEJMoa1903076

[39] Wollin L , Togbe D , Ryffel B . Effects of nintedanib in an animal model of liver fibrosis. Biomed Res Int. 2020;2020:3867198. 32337244 10.1155/2020/3867198 PMC7150695

[40] Singh B , Sims H , Trueheart I , et al. A phase I clinical trial to assess safety and tolerability of injectable collagenase in women with symptomatic uterine fibroids. Reprod Sci. 2021. 10.1007/s43032-021-00573-8. PMC8346429 33914296

[41] Moutos FT , Estes BT , Guilak F . Multifunctional hybrid three‐dimensionally woven scaffolds for cartilage tissue engineering. Macromol Biosci. 2010;10(11):1355–1364. 20857388 10.1002/mabi.201000124 PMC3214627

[42] Rhim JS . Generation of immortal human prostate cell lines for the study of prostate cancer. Methods Mol Med. 2003;81:69–77. 12725115 10.1385/1-59259-372-0:69

[43] Malik M , Webb J , Catherino WH . Retinoic acid treatment of human leiomyoma cells transformed the cell phenotype to one strongly resembling myometrial cells. Clin Endocrinol (Oxf). 2008;69(3):462–470. 18248652 10.1111/j.1365-2265.2008.03207.x PMC2610401

[44] Joseph DS , Malik M , Nurudeen S , Catherino WH . Myometrial cells undergo fibrotic transformation under the influence of transforming growth factor beta‐3. Fertil Steril. 2010;93(5):1500–1508. 19328471 10.1016/j.fertnstert.2009.01.081

[45] Malik M , Britten J , Borahay M , Segars J , Catherino WH . Simvastatin, at clinically relevant concentrations, affects human uterine leiomyoma growth and extracellular matrix production. Fertil Steril. 2018;110(7):1398–1407. e1391. 30503138 10.1016/j.fertnstert.2018.07.024

[46] Haller ST , Kennedy DJ , Shidyak A , et al. Monoclonal antibody against marinobufagenin reverses cardiac fibrosis in rats with chronic renal failure. Am J Hypertens. 2012;25(6):690–696. 22378033 10.1038/ajh.2012.17 PMC3355226

[47] Ma S , Meng Z , Chen R , Guan K‐L . The Hippo pathway: biology and pathophysiology. Annu Rev Biochem. 2019;88:577–604. 30566373 10.1146/annurev-biochem-013118-111829

[48] Islam MS , Catherino WH , Protic O , et al. Role of activin‐A and myostatin and their signaling pathway in human myometrial and leiomyoma cell function. J Clin Endocrinol Metab. 2014;99(5):E775–E785. 24606069 10.1210/jc.2013-2623 PMC4010707

[49] Arici A , Sozen I . Transforming growth factor‐beta3 is expressed at high levels in leiomyoma where it stimulates fibronectin expression and cell proliferation. Fertil Steril. 2000;73(5):1006–1011. 10785229 10.1016/s0015-0282(00)00418-0

[50] Norian JM , Malik M , Parker CY , et al. Transforming growth factor beta3 regulates the versican variants in the extracellular matrix‐rich uterine leiomyomas. Reprod Sci. 2009;16(12):1153–1164. 19700613 10.1177/1933719109343310 PMC2857408

[51] Ciarmela P , Bloise E , Gray PC , et al. Activin‐A and myostatin response and steroid regulation in human myometrium: disruption of their signalling in uterine fibroid. J Clin Endocrinol Metab. 2011;96(03):755–765. 21177794 10.1210/jc.2010-0501 PMC3047220

[52] Protic O , Toti P , Islam MS , et al. Possible involvement of inflammatory/reparative processes in the development of uterine fibroids. Cell Tissue Res. 2015;364(2):415–427. 26613601 10.1007/s00441-015-2324-3

[53] Protic O , Islam MS , Greco S , et al. Activin A in inflammation, tissue repair, and fibrosis: possible role as inflammatory and fibrotic mediator of uterine fibroid development and growth. Semin Reprod Med. 2017;35(06):499–509. 29100238 10.1055/s-0037-1607265

[54] Gaedeke J , Boehler T , Budde K , Neumayer H‐H , Peters H . Glomerular activin A overexpression is linked to fibrosis in anti‐Thy1 glomerulonephritis. Nephrol Dial Transplant. 2005;20(2):319–328. 15673690 10.1093/ndt/gfh653

[55] Leppert PC , Jayes FL , Segars JH . The extracellular matrix contributes to mechanotransduction in uterine fibroids. Obstet Gynecol Int. 2014;2014:783289. 25110476 10.1155/2014/783289 PMC4106177

[56] Flevaris P , Vaughan D . The role of plasminogen activator inhibitor type‐1 in fibrosis. Semin Thromb Hemost. 2017;43(02):169–177. 27556351 10.1055/s-0036-1586228

[57] Islam MS , Giampieri F , Janjusevic M , et al. An anthocyanin rich strawberry extract induces apoptosis and ROS while decreases glycolysis and fibrosis in human uterine leiomyoma cells. Oncotarget. 2017;8(14):23575. 28212568 10.18632/oncotarget.15333 PMC5410328

[58] Jayes FL , Liu B , Feng L , Aviles‐Espinoza N , Leikin S , Leppert PC . Evidence of biomechanical and collagen heterogeneity in uterine fibroids. PLoS ONE. 2019;14(4):e0215646. 31034494 10.1371/journal.pone.0215646 PMC6488189

[59] Noguchi S , Saito A , Mikami Y , et al. TAZ contributes to pulmonary fibrosis by activating profibrotic functions of lung fibroblasts. Sci Rep. 2017;7:42595. 28195168 10.1038/srep42595 PMC5307361

[60] Szeto SG , Narimatsu M , Lu M , et al. YAP/TAZ are mechanoregulators of TGF‐β‐Smad signaling and renal fibrogenesis. J Am Soc Nephrol. 2016;27(10):3117–3128. 26961347 10.1681/ASN.2015050499 PMC5042658

[61] Camargo FD , Gokhale S , Johnnidis JB , et al. YAP1 increases organ size and expands undifferentiated progenitor cells. Curr Biol. 2007;17(23):2054–2060. 17980593 10.1016/j.cub.2007.10.039

[62] Ducharme MT , Purdy MP , Haak AJ , et al. Increased baseline YAP/TAZ nuclear localization and altered transcriptional activity in uterine fibroids. FASEB J. 2019;33(1_supplement):712.711–712.711.

[63] Lasky JA , Ortiz LA , Tonthat B , et al. Connective tissue growth factor mRNA expression is upregulated in bleomycin‐induced lung fibrosis. Am J Physiol. 1998;275(2):L365–371. 9700098 10.1152/ajplung.1998.275.2.L365

[64] Sun Z , Wang Y , Cai Z , Chen P , Tong X , Xie D . Involvement of Cyr61 in growth, migration, and metastasis of prostate cancer cells. Br J Cancer. 2008;99(10):1656–1667. 18941464 10.1038/sj.bjc.6604712 PMC2584944

[65] Serini G , Bochaton‐Piallat M‐L , Ropraz P , et al. The fibronectin domain ED‐A is crucial for myofibroblastic phenotype induction by transforming growth factor‐beta1. J Cell Biol. 1998;142(3):873–881. 9700173 10.1083/jcb.142.3.873 PMC2148176

[66] Bukong TN , Maurice SB , Chahal B , Schaeffer DF , Winwood PJ . Versican: a novel modulator of hepatic fibrosis. Lab Invest. 2016;96(3):361–374. 26752747 10.1038/labinvest.2015.152

[67] Andersson‐Sjöland A , Hallgren O , Rolandsson S , et al. Versican in inflammation and tissue remodeling: the impact on lung disorders. Glycobiology. 2015;25(3):243–251. 25371494 10.1093/glycob/cwu120 PMC4310351

[68] Afrin S , Islam MS , Patzkowsky K , et al. Simvastatin ameliorates altered mechanotransduction in uterine leiomyoma cells. Am J Obstet Gynecol. 2020;223(5):733.e731–733.e714. 32417359 10.1016/j.ajog.2020.05.012 PMC7609626

[69] Wang Z , Wu Y , Wang H , et al. Interplay of mevalonate and Hippo pathways regulates RHAMM transcription via YAP to modulate breast cancer cell motility. Proc Natl Acad Sci U S A. 2014;111(1):E89–E98. 24367099 10.1073/pnas.1319190110 PMC3890879

