# Supplementary figures and images for: Extracellular matrix and Hippo signaling as therapeutic targets of antifibrotic compounds for uterine fibroids
Source: Clin Transl Med. 2021 Jul 4;11(7):e475. doi: 10.1002/ctm2.475 (PMC8255059; doi:10.1002/ctm2.475)

## 24-48 hrs post injection

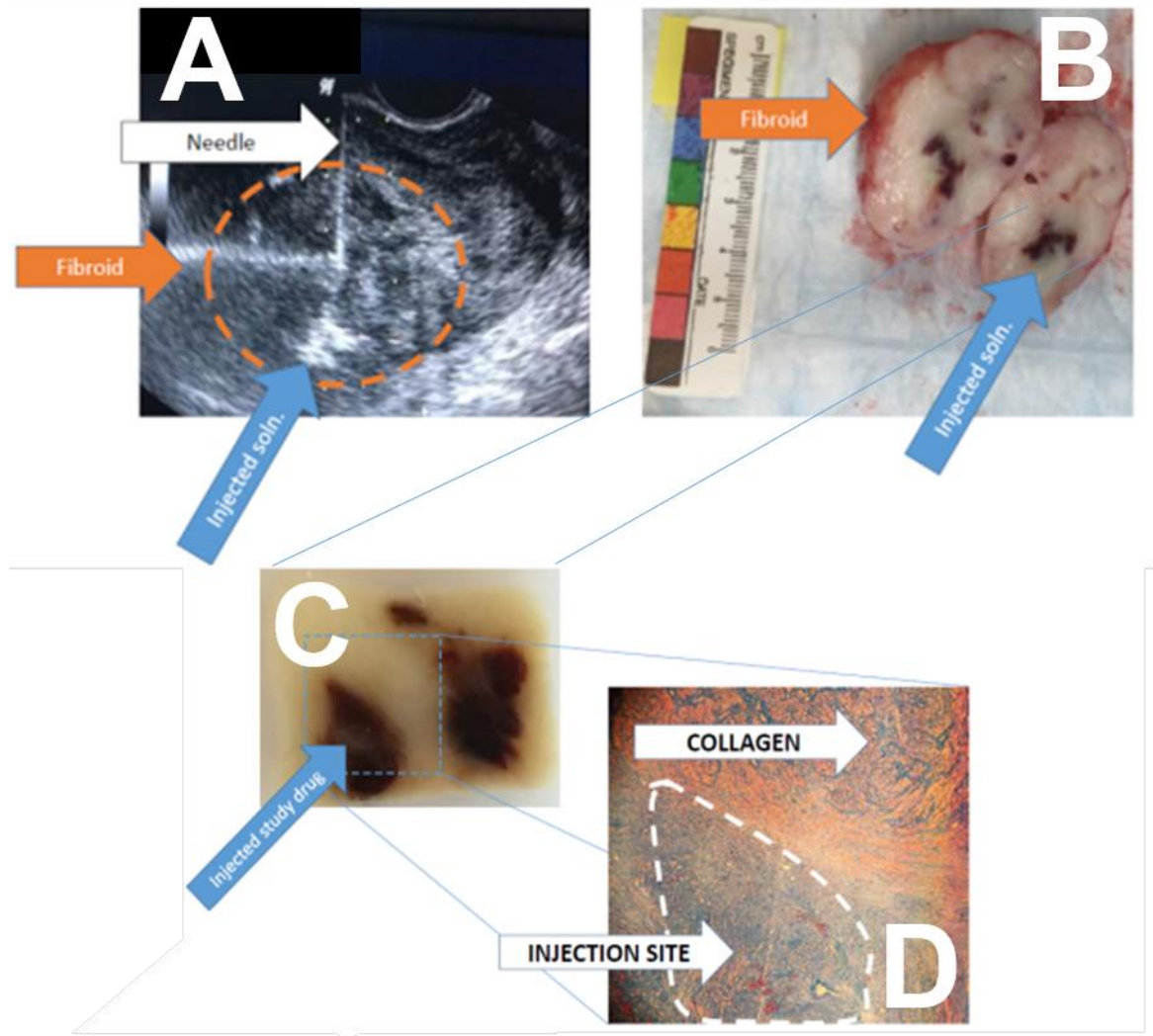

Supplement: Supplementary file 1 — SUPPORTING INFORMATION [file CTM2-11-e475-s006.pdf]

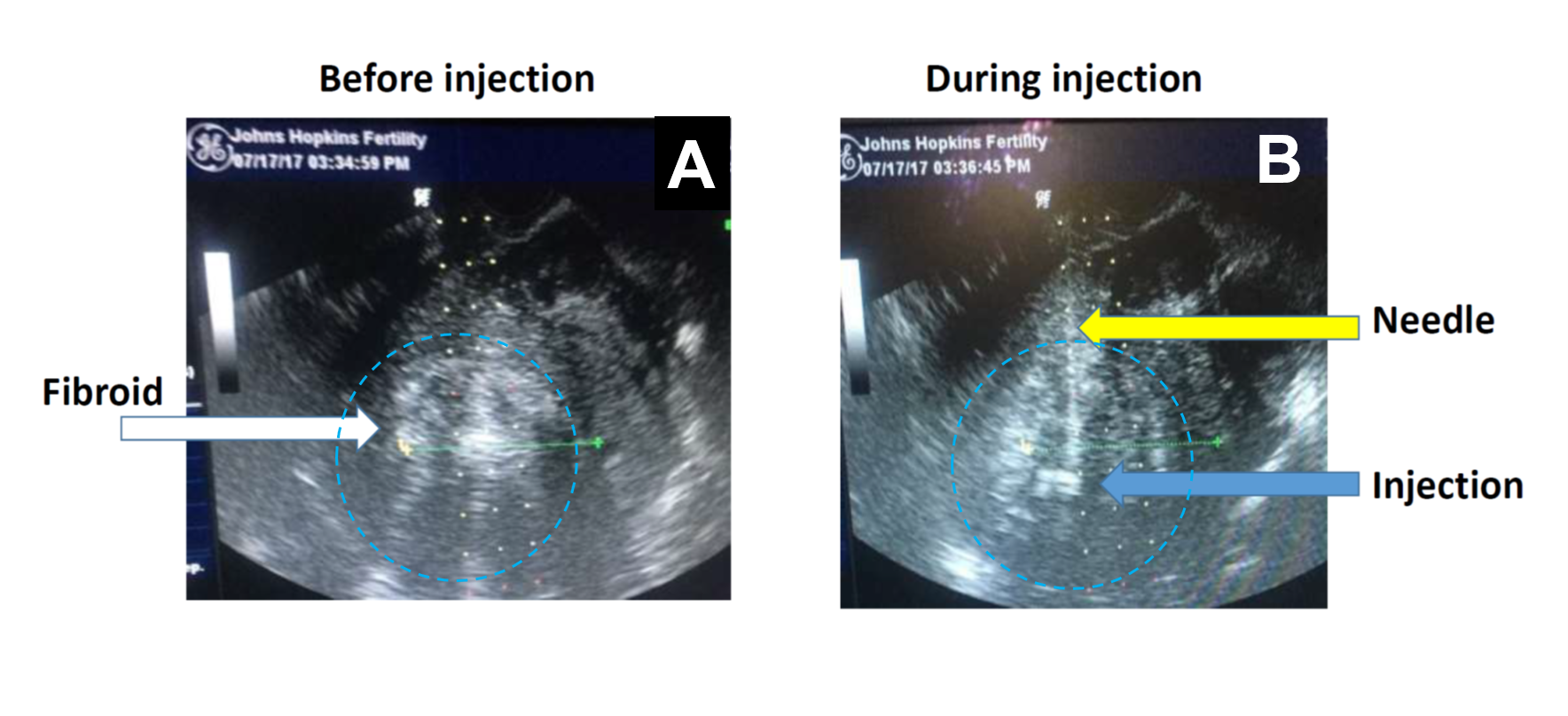

Supplement: Supplementary file 2 — SUPPORTING INFORMATION [file CTM2-11-e475-s003.png]

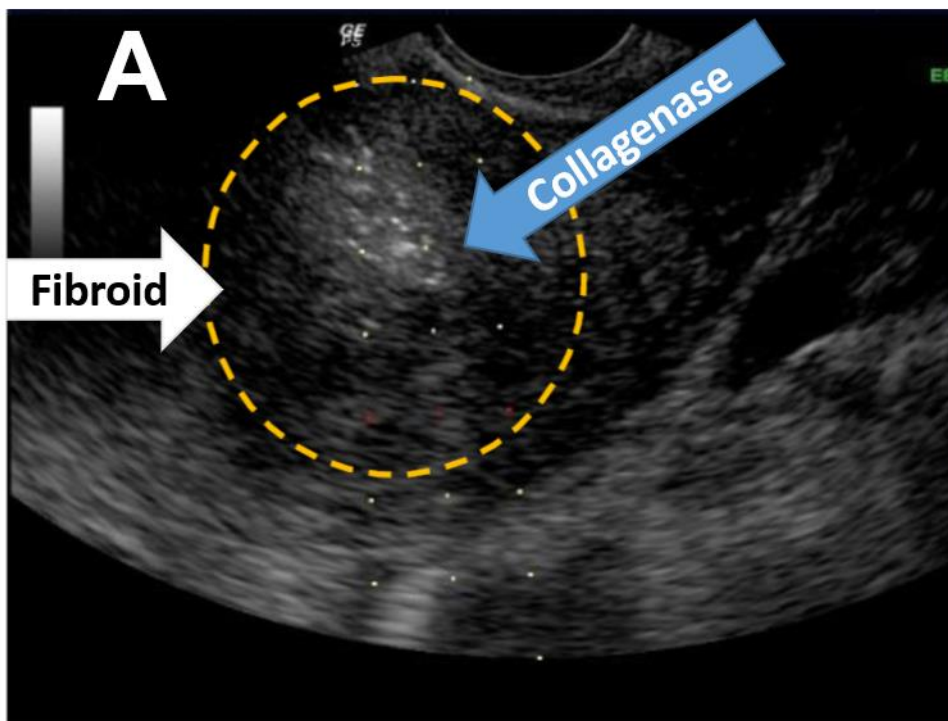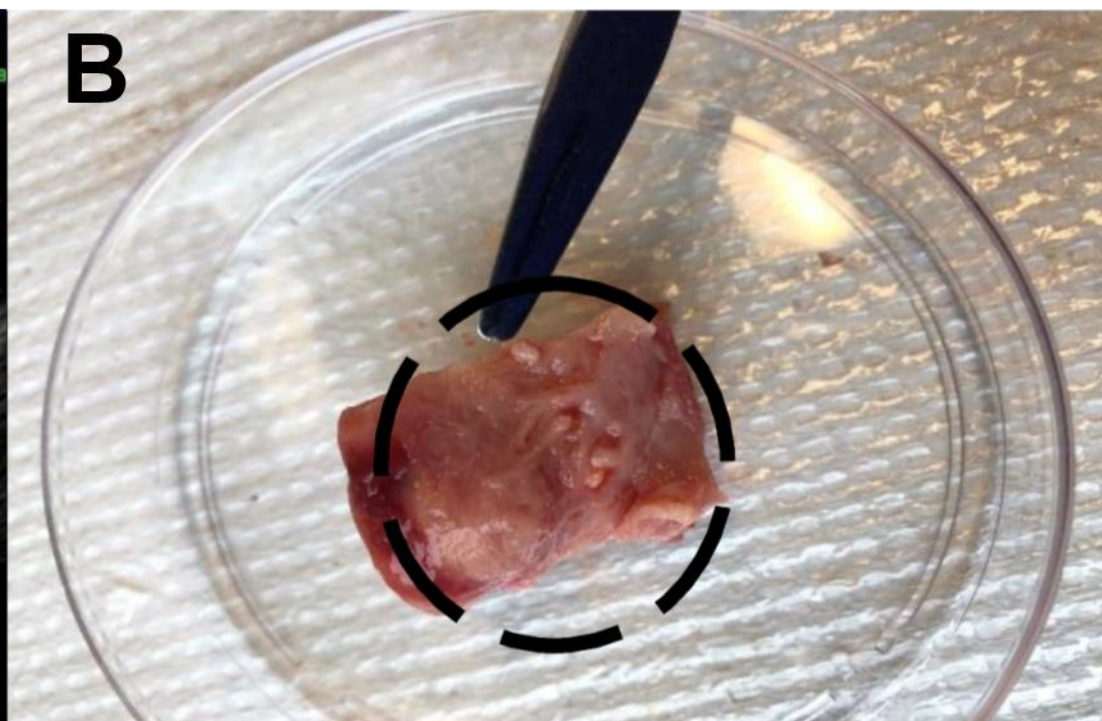

Supplement: Supplementary file 3 — SUPPORTING INFORMATION [file CTM2-11-e475-s011.pdf]

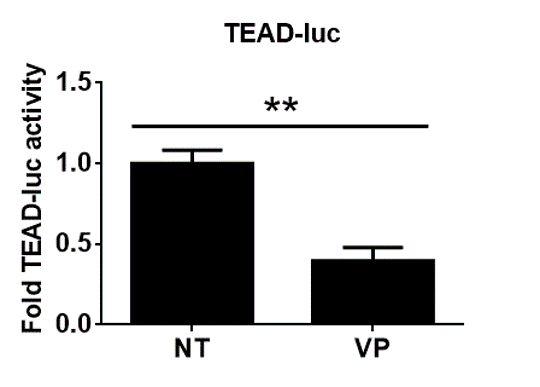

Supplement: Supplementary file 4 — SUPPORTING INFORMATION [file CTM2-11-e475-s009.png]

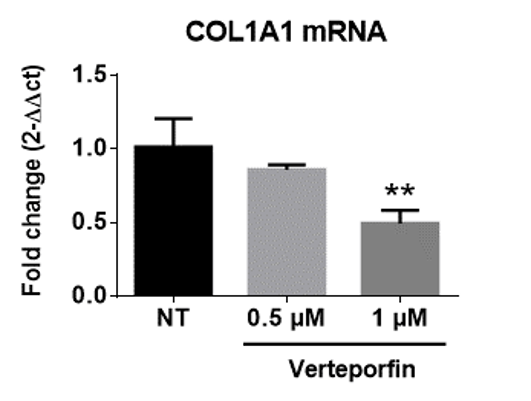

Supplement: Supplementary file 5 — SUPPORTING INFORMATION [file CTM2-11-e475-s008.png]

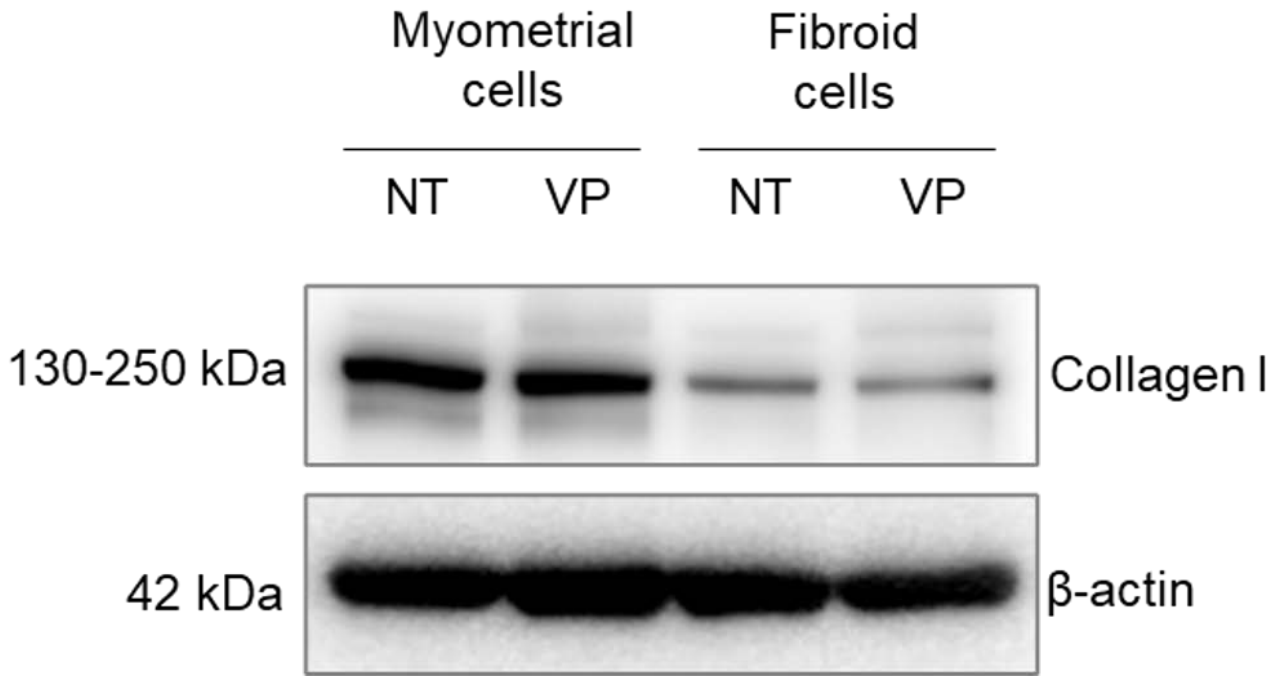

Supplement: Supplementary file 6 — SUPPORTING INFORMATION [file CTM2-11-e475-s001.PDF]

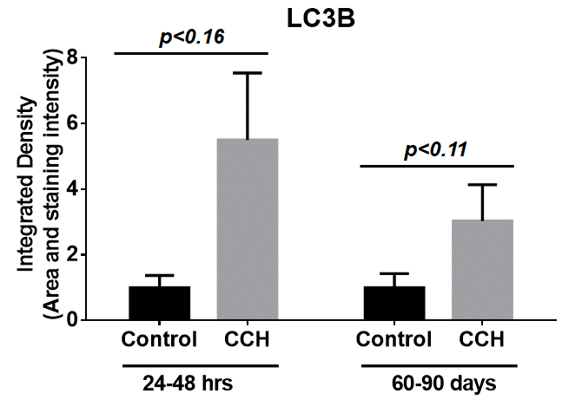

Supplement: Supplementary file 8 — SUPPORTING INFORMATION [file CTM2-11-e475-s005.png]
