# Supplementary material for: Extracellular matrix and Hippo signaling as therapeutic targets of antifibrotic compounds for uterine fibroids
Source: Clin Transl Med. 2021 Jul 4;11(7):e475. doi: 10.1002/ctm2.475 (PMC8255059; doi:10.1002/ctm2.475)
Supplement: Supplementary file 7 — SUPPORTING INFORMATION [file CTM2-11-e475-s004.pdf]

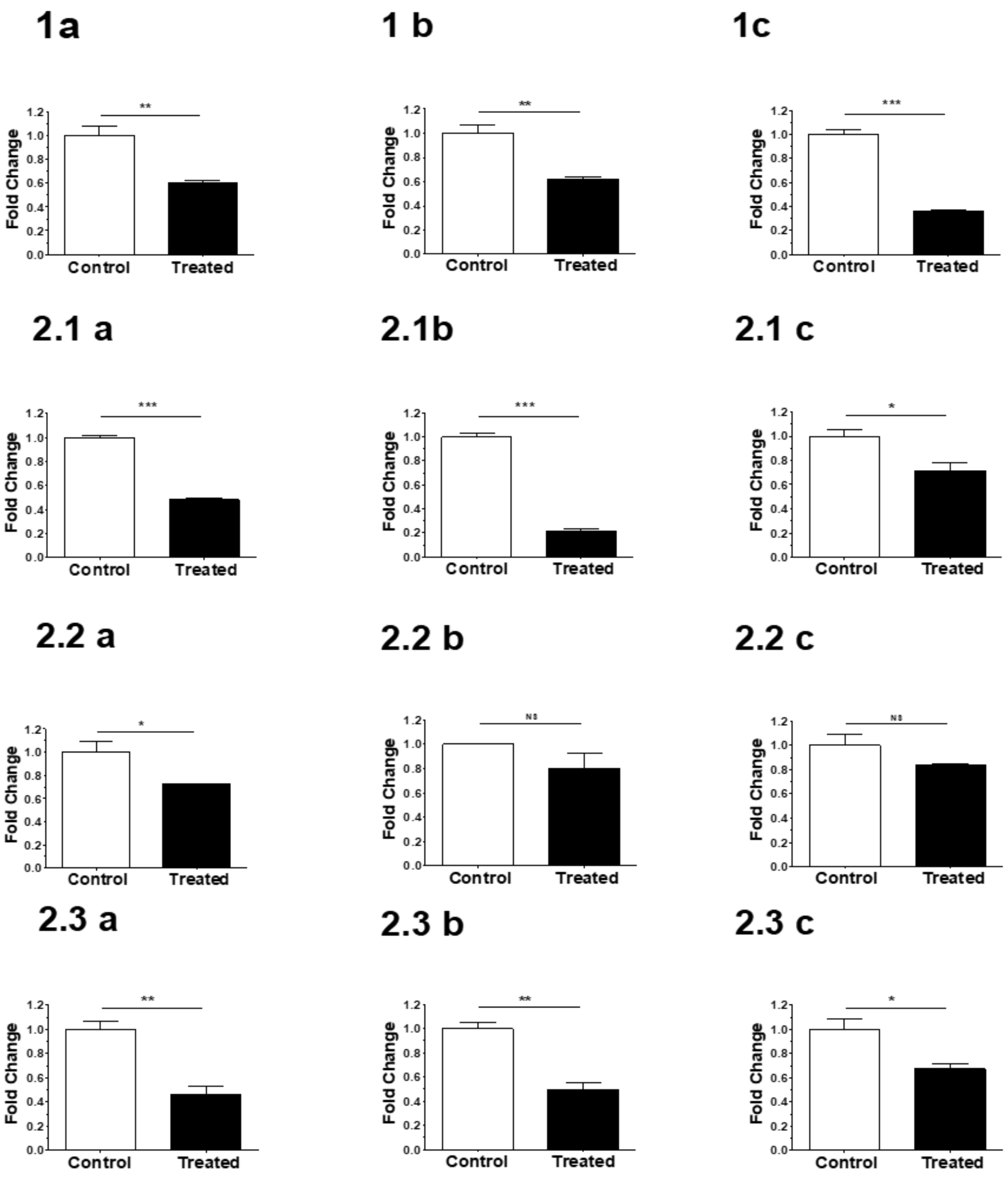

1a
1 b
1c
2.1 a
2.1b
2.1 c
2.2 a
2.2 b
2.2 c
2.3 a
2.3 b
2.3 c
Fold Change
Control
Treated
0.0
0.2
0.4
0.6
0.8
1.0
1.2
*
**
***
NS
