# Supplementary material for: Extracellular matrix and Hippo signaling as therapeutic targets of antifibrotic compounds for uterine fibroids
Source: Clin Transl Med. 2021 Jul 4;11(7):e475. doi: 10.1002/ctm2.475 (PMC8255059; doi:10.1002/ctm2.475)
Supplement: Supplementary file 10 — SUPPORTING INFORMATION [file CTM2-11-e475-s002.docx]

**Table S2.** List of primary antibodies.

| **Primary antibodies** | **Cat no.** | **Company** | **Size (kDa)** | **Host/Isotype** | **WB dil.** | **IF dil.** |
| --- | --- | --- | --- | --- | --- | --- |
| α-SMA | A2547 | Sigma Aldrich | ~ 42 | Mouse | - | 1: 1000 |
| Fibronectin | ab6584 | Abcam | 238-268 | Rabbit | 1: 10000 | - |
| PAI-1 | 11907 | Cell Signaling Technology | 48 | Rabbit IgG | 1: 1000 | - |
| PCNA | 13110 | Cell Signaling Technology | 36 | Rabbit IgG | 1: 1000 | 1: 100 |
| Phospho-FAK | 3283 | Cell Signaling Technology | 125 | Rabbit IgG | 1: 500 | - |
| Phospho-p44/42 MAPK (ERK1/2) | 4377 | Cell Signaling Technology | 42, 44 | Rabbit IgG | 1: 500 | - |
| Phospho-SMAD2 | 3108 | Cell Signaling Technology | 60 | Rabbit IgG | 1: 500 | - |
| Phospho-YAP | 4911 | Cell Signaling Technology | 65-78 | Rabbit | 1: 1000 | 1: 100 |
| YAP | 4912 | Cell Signaling Technology | 65-78 | Rabbit | 1: 500 | - |
| YAP/TAZ (D24E4) | 8418 | Cell Signaling Technology | 55, 78 | Rabbit IgG | - | 1: 50 |
| Non-phospho-YAP | 29495 | Cell Signaling Technology | 65-78 | Rabbit IgG | 1: 500 | - |
| Collagen I | PA5-29569 | Invitrogen | 130-250 | Rabbit / IgG | 1: 600 | - |
| LC3B | NB600-1384 | Novus Biologicals | 14.6 | Rabbit | - | 1: 250 |
| β-Actin | A3854 | Sigma Aldrich | 42 | Mouse IgG | 1:50,000 | - |
