# Supplementary material for: Extracellular matrix and Hippo signaling as therapeutic targets of antifibrotic compounds for uterine fibroids
Source: Clin Transl Med. 2021 Jul 4;11(7):e475. doi: 10.1002/ctm2.475 (PMC8255059; doi:10.1002/ctm2.475)
Supplement: Supplementary file 11 — SUPPORTING INFORMATION [file CTM2-11-e475-s007.docx]

**Table S3** List of primers.

| **Gene name** | **RefSeq** | **Product length** | **Direction** | **Sequence (5'->3')** | **Length** | **Tm** | **GC%** |
| --- | --- | --- | --- | --- | --- | --- | --- |
| CCND1 | NM_053056.2 | 135 | Forward | GCTGCGAAGTGGAAACCATC | 20 | 59.83 | 55.00 |
|  |  |  | Reverse | CCTCCTTCTGCACACATTTGAA | 22 | 59.11 | 45.45 |
| CTGF | NM_001901.3 | 146 | Forward | CAAGGGCCTCTTCTGTGACT | 20 | 59.31 | 55.00 |
|  |  |  | Reverse | ACGTGCACTGGTACTTGCAG | 20 | 60.88 | 55.00 |
| CYR61 | NM_001554.5 | 136 | Forward | CAGCTGACCAGGACTGTGAA | 20 | 59.61 | 55.00 |
|  |  |  | Reverse | TGTAGAAGGGAAACGCTGCT | 20 | 59.31 | 50.00 |
| SAV1 | XM_017021568.2 | 142 | Forward | CCTGTGCTCCTAGTGTACCTC | 21 | 59.25 | 57.14 |
|  |  |  | Reverse | GCGTAAACCTGAAGCCAGTC | 20 | 59.20 | 55.00 |
| MST1 | NM_006282.5 | 75 | Forward | AGTGCCAAAGGAGTGTCAATAC | 22 | 58.59 | 45.45 |
|  |  |  | Reverse | GGATTCCTGGCGTTTCAGTTTC | 22 | 60.10 | 50.00 |
| LATS1 | NM_004690.4 | 163 | Forward | CAAGATCCTCGACGAGAGCA | 20 | 59.26 | 55.00 |
|  |  |  | Reverse | CACTTTCTCCTAGTGGCGGG | 20 | 60.11 | 60.00 |
| FN1 | NM_212482.3 | 142 | Forward | CCATAAAGGGCAACCAAGAG | 20 | 56.38 | 50.00 |
|  |  |  | Reverse | AAACCAATTCTTGGAGCAGG | 20 | 56.20 | 45.00 |
| VCAN | NM_001126336.3 | 80 | Forward | GAAAAGTCAGCCTACCTTATC | 21 | 53.73 | 42.86 |
|  |  |  | Reverse | GATGCGGAGAAATTCACTGG | 20 | 56.88 | 50.00 |
| COL1A1 | NM_000088.4 | 162 | Forward | GGTGAACAGGGTGTTCCTGGAGAC | 24 | 64.88 | 58.33 |
|  |  |  | Reverse | AGCATCACCCTTAGCACCATCGTT | 24 | 64.50 | 50.00 |
| INHBA | NM_002192.4 | 156 | Forward | CATCACCTTTGCCGAGTCAG | 20 | 58.92 | 55.00 |
|  |  |  | Reverse | AGACGGATGGTGACTTTGGT | 20 | 58.94 | 50.00 |
| ACVR2A | NM_001278579.2 | 299 | Forward | CGTTCGCCGTCTTTCTTATC | 20 | 57.03 | 50.00 |
|  |  |  | Reverse | GCCCTCACAGCAACAAAAGT | 20 | 59.25 | 50.00 |
| ACVR2B | NM_001106.4 | 189 | Forward | AGACACGGGAGTGCATCTACT | 21 | 60.62 | 52.38 |
|  |  |  | Reverse | GCCTATCGTAGCAGTTGAAGTC | 22 | 58.88 | 50.00 |
| ACVR1B | NM_020328.4 | 227 | Forward | TTCTTCCCCCTTGTTGTCCT | 20 | 58.47 | 50.00 |
|  |  |  | Reverse | AGGCAGTAGAAGGGCTTTCC | 20 | 59.38 | 55.00 |
| SMAD2 | NM_001003652.4 | 259 | Forward | ATGTCGTCCATCTTGCCATT | 20 | 57.57 | 45.00 |
|  |  |  | Reverse | GTCCCCAAATTTCAGAGCAA | 20 | 56.21 | 45.00 |
| PAI-1 | NM_000602.4 | 127 | Forward | GAGAACCTGGGAATGACCGAC | 21 | 60.41 | 57.14 |
|  |  |  | Reverse | TGCCACTCTCGTTCACCTCG | 20 | 62.14 | 60.00 |
| ITGB1 | NM_002211.4 | 107 | Forward | GAAGGGTTGCCCTCCAGA | 18 | 58.50 | 61.11 |
|  |  |  | Reverse | GCTTGAGCTACTCTGCTGTT | 20 | 57.91 | 50.00 |
| ITGA6 | NM_000210.4 | 113 | Forward | TTGAATATACTGCTAACCCCG | 21 | 55.19 | 42.86 |
|  |  |  | Reverse | TCGAAACTGAACTCTTGAGAATAG | 24 | 56.50 | 37.50 |
| FAK | XM_017013688.2 | 103 | Forward | GCGGCCCAGGTTTACTGAA | 19 | 60.30 | 57.89 |
|  |  |  | Reverse | GGCCTGTCTTCTGGACTCCA | 20 | 61.19 | 60.00 |
| AKAP13 | NM_006738.6 | 87 | Forward | CAGTGATGACATGGACAG | 18 | 52.37 | 50.00 |
|  |  |  | Reverse | TCGGTGGATGAACTGGATC | 19 | 56.53 | 52.63 |
| RPLP0 | NM_001002.4 | 265 | Forward | GCGACCTGGAAGTCCAACT | 19 | 59.63 | 57.89 |
|  |  |  | Reverse | GGTCCTCCTTGGTGAACAC | 19 | 57.68 | 57.89 |
